# CLRN1 Variants in Müller Cells Cause Mitochondrial Dysfunction in USH3A Retinal Organoids

**DOI:** 10.1002/cns.71068

**Published:** 2026-08-03

**Authors:** Rui Zhang, Xinbo Ji, Han Yu, Jingwen Xu, Yu Wang, Ping Sun, Yingxin Wang, Yao Tang, Zexin Zhan, Yichang Jiao, Didi Shan, Pengfei Lin, Dong‐dong Wang, Yuying Zhao, Xianyang Liu, Chuanzhu Yan, Jianqiao Li, Mingfeng Li, Fuchen Liu, Shengping Hou

**Affiliations:** ^1^ Department of Ophthalmology Qilu Hospital of Shandong University Jinan China; ^2^ Department of Neurology, Research Institute of Neuromuscular and Neurodegenerative Diseases Qilu Hospital of Shandong University, Shandong Key Laboratory of Mitochondrial Medicine and Rare Diseases Jinan China; ^3^ Prenatal Diagnostic Center of Obstetrics and Department of Gynecology Qilu Hospital of Shandong University Jinan China; ^4^ Beijing Institute of Ophthalmology, Beijing Tongren Eye Center, Beijing Tongren Hospital Capital Medical University, Beijing Ophthalmology & Visual Sciences Key Laboratory Beijing China; ^5^ Qingdao Municipal Key Laboratory of Mitochondria Medicine Qingdao China; ^6^ Department of Pharmacology, Innovation Center for Brain Medical Sciences, Tongji Medical College Huazhong University of Science and Technology, the Key Laboratory for Drug Target Researches and Pharmacodynamic Evaluation of Hubei Province Wuhan China

## Abstract

**Background:**

Usher syndrome 3A (USH3A), caused by mutations in the *CLRN1* gene, leads to retinitis pigmentosa and sensorineural hearing loss. While *CLRN1*'s role in inner ear pathology is established, its contribution to retinal degeneration remains poorly understood.

**Methods:**

Retinal organoids derived from a USH3A patient were analyzed using single‐cell RNA sequencing and multi‐electrode array recording. *CLRN1* expression was mapped in human fetal retina and organoids. We assessed the structural, transcriptional, and functional impact of *CLRN1* variants on Müller cells and photoreceptors, and evaluated idebenone as a potential targeted therapy.

**Results:**

*CLRN1* was specifically expressed in Müller cells. *CLRN1* variants induced severe retinal degeneration, characterized by outer nuclear layer thinning, impaired photoreceptor gene expression, activated apoptosis, and diminished electrophysiological function. Mechanistically, these variants caused mitochondrial dysfunction in Müller cells, which triggered secondary mitochondrial impairment, oxidative stress, and apoptosis in photoreceptors. Idebenone treatment partially rescued these deficits.

**Conclusions:**

*CLRN1*‐related mitochondrial impairment in Müller cells contributes to the pathogenesis of retinitis pigmentosa in USH3A. These findings identify Müller cell mitochondrial dysfunction as a key disease mechanism and highlight potential therapeutic targets.

## Graphical abstract

We generated retinal organoids from a patient with USH3A and performed single‐cell RNA sequencing. CLRN1 was specifically expressed in Müller cells, where its variants led to mitochondrial dysfunction and photoreceptor degeneration.

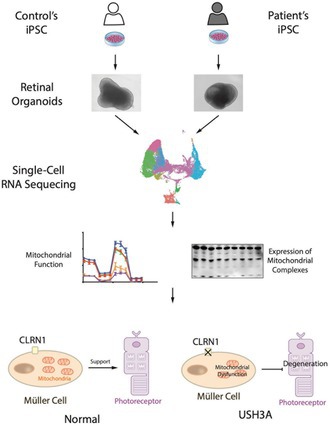

## 1 Introduction

Usher syndrome (USH) is an auto‐recessive disorder, characterized by retinitis pigmentosa and sensorineural hearing loss, with or without the involvement of other systems. This rare disease affects approximately 2 in 100,000 people worldwide [1]. The prevalence of USH type 3A (USH3A), a relatively mild subtype of the syndrome, varies significantly, accounting for 1.7% to 40% of USH cases [2, 3]. USH3A is marked by postlingual progressive hearing loss, variable vestibular dysfunction, and late‐onset retinitis pigmentosa. The ocular symptoms include nyctalopia, constriction of visual fields, and a gradual loss of central visual acuity. Despite the clinical significance of USH3A, the precise mechanisms through which it causes retinitis pigmentosa remain poorly understood, and no effective treatment currently exists.

*CLRN1*, which encodes Clarin‐1, is the gene responsible for causing USH3A. Previous studies using *CLRN1* knockout mouse models have shown that the loss of Clarin‐1 disrupts the cytoskeleton and disturbs ion balance in hair cells [4]. Clarin‐1 is selectively expressed in inner hair cells of the ear, where it co‐localizes with other USH proteins, including PCDH15 (associated with USH1F), to form a complex that regulates mechanotransduction channels [5]. Moreover, Clarin‐1 interacts with the CaV_1.3_ Ca^2+^ channel complex via direct interaction with HARMONIN (encoded by the *USH1C* gene), thereby influencing Ca^2+^ currents and membrane capacitance [4, 6]. Clarin‐1 also plays a critical role in maintaining cytoskeletal homeostasis by directly interacting with F‐actin [7] and regulating ciliary oscillation [4].

While the function of Clarin‐1 in hair cells has been extensively studied, its role in the retina remains unclear. Notably, *CLRN1* knockout mice exhibit hearing and vestibular dysfunction but display no retinal abnormalities, including normal retinal layer thickness, normal a‐ and b‐wave amplitudes in electroretinograms, and normal retinal markers like glial fibrillary acidic protein (GFAP) and Vimentin (VIM) [8]. This discrepancy is believed to result from the differential expression of *CLRN1* between mice and humans. In mice, *CLRN1* mRNA expression is detectable in the retina at postnatal Day 7 (P7) but diminishes and becomes undetectable by Week 4 and adulthood [9]. In contrast, *CLRN1* mRNA is continuously expressed in the human retina, suggesting a more significant role in maintaining retinal structure and function in humans [8]. Thus, human‐derived models are essential to study the role of Clarin‐1 in the retina.

In this study, we report a case of a patient diagnosed with USH3A, harboring two heterozygous variants in *CLRN1*, presenting with sensorineural deafness, retinitis pigmentosa, and vestibular dysfunction. These two variants, inherited from his parents, result in reduced expression of Clarin‐1. To investigate the impact of these variants, we generated induced pluripotent stem cells (iPSCs) from the patient's skin fibroblasts and differentiated them into retinal organoids. These retinal organoids, containing various retinal cell types including photoreceptors, bipolar cells, amacrine cells, horizontal cells, ganglion cells, Müller cells, astrocytes, and retinal pigment epithelium (RPE) cells, were cultured until Day 150 and analyzed using single‐cell RNA sequencing. Our findings revealed that the patient's retinal organoids exhibited features of retinal degeneration, including a thinned outer nuclear layer (ONL), reduced phototransduction gene expression, and diminished electrophysiological function. Additionally, variants in *CLRN1* led to reduced expression of mitochondrial complex genes in Müller cells and caused mitochondrial dysfunction. These deficiency in Müller cells caused loss of mitochondrial complexes and photoreceptor‐specific markers and apoptotic cell death in photoreceptors. These observations highlight the critical role of Clarin‐1 in maintaining mitochondrial function in Müller cells and suggest that mitochondrial dysfunction in these cells may contribute to retinitis pigmentosa in USH3A.

## 2 Results

### 2.1 Clinical Report

**FIGURE 1:**
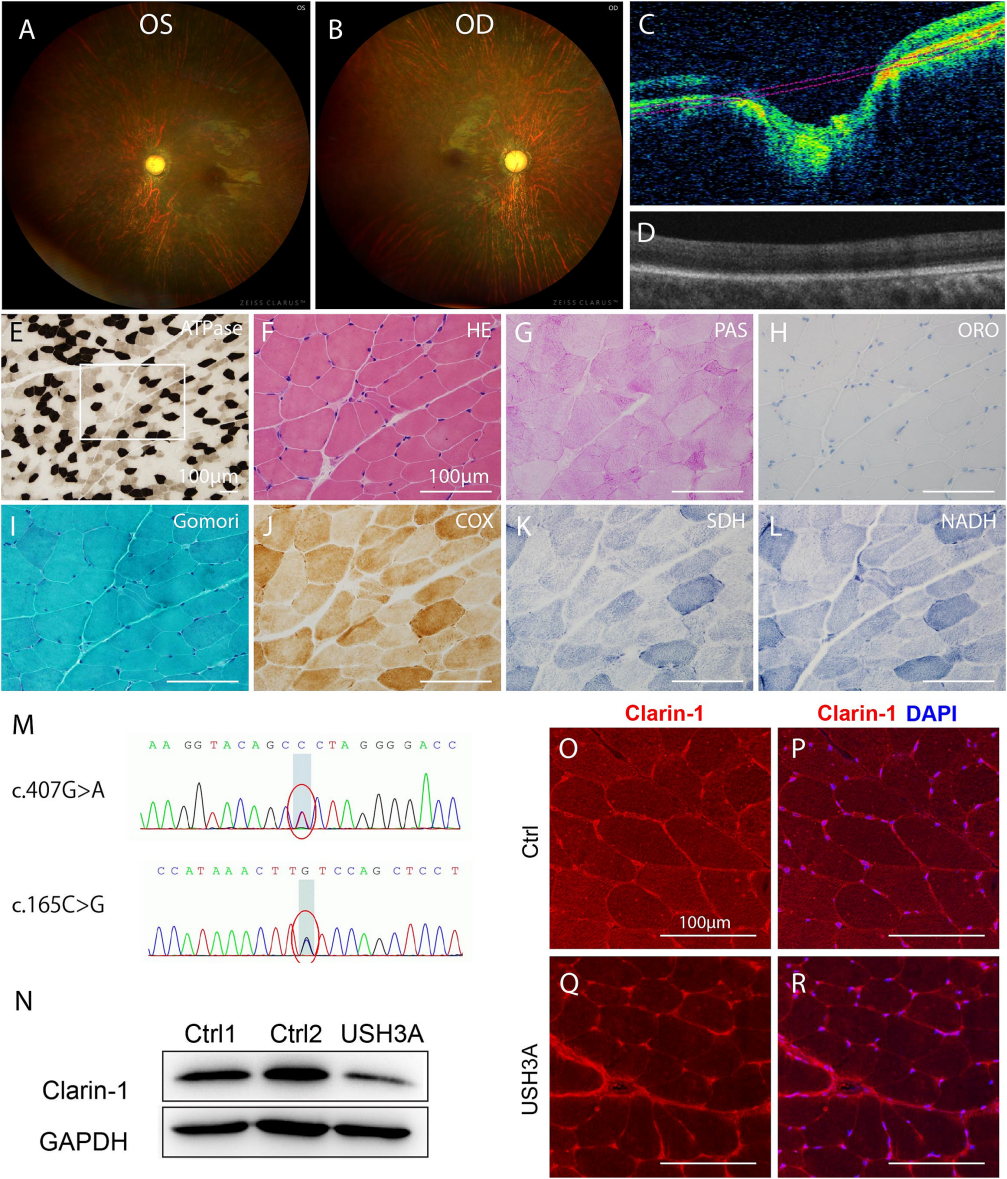
Clinical information about the patient. (A, B) Fundus photography of the USH3A patient illustrated retinitis pigmentosa. (C, D) OCT of the patient found reduced thickness in the neural retina and enlarged fovea. (E–L) Muscle pathologies of the patient. ATPase staining (pH = 4.3) (E), HE staining (F), PAS staining for glycogen(G), ORO staining for lipid (H), modified Gomori staining (I), COX (J), SDH (K) and NADH (L) staining of the muscle specimens illustrated relatively normal muscle histology. Scale bar: 100 μm. (M) Whole exosome sequencing found 2 heterozygous variants in *CLRN1*. (N) Western blot of Clarin‐1 in the muscles of 2 normal controls (Ctrl) and the USH3A patient. (O–R) Immunofluorescence of frozen muscle sections found reduced expression of Clarin‐1 in the patient. Scale bar: 100 μm.

A 26‐year‐old male patient presented to our clinic with a history of progressive vision loss, hearing impairment, slurred speech, and difficulty walking, symptoms that began at the age of 14. Neurological examination revealed nystagmus, ataxia, hypertonia, and a scissor gait. His visual acuity was 0.5/0.5 and could not be corrected. Fundus photography confirmed the presence of retinitis pigmentosa (Figure 1A,B). Optical coherence tomography showed a significant reduction in the thickness of the neural retina (Figure 1C,D). Pattern visual evoked potential testing indicated delayed p100 peak time and reduced amplitude bilaterally, suggestive of optic nerve conduction abnormalities. Pure tone audiometry identified sensorineural hearing loss with prolonged latency. The cold‐hot water test indicated vestibular dysfunction. All other members of the patient's family were healthy.

A muscle biopsy was performed to investigate potential muscular disorders, revealing nearly normal muscle tissue with only slight variation in muscle fiber size (Figure 1E–L). Consistent with these findings, electromyography did not detect any muscular abnormalities but did reveal sensory nerve dysfunction. Whole‐exome sequencing identified heterozygous variants in the *CLRN1* gene: c.165C>G (p.D55E) in exon 1 and c.407G>A (p.G136E) in exon 2, inherited from his father and mother, respectively (Figure 1M). The c.165C>G (p.D55E) variant was identified as a de novo mutation, while the variant affecting glycine at position 136, associated with USH3A, has been previously reported [10, 11]. These 2 sites were both highly conserved among mammals (https://www.uniprot.org/; Figure S1).

Western blot analysis demonstrated that Clarin‐1 is expressed in human skeletal muscle, with both *CLRN1* mRNA and Clarin‐1 protein levels significantly reduced in the USH3A patient compared with two control subjects (Figures 1N and S2A). Next, HEK293 cells were transfected with vector controls, wild‐type *CLRN1* (hereafter referred to as WT), *CLRN1* with the c.165C>G variant (hereafter referred to as E1), and *CLRN1* with the c.407G>A variant (hereafter referred to as E2). The mRNA levels of *CLRN1* were reduced in cells harboring the E1 and E2 variants (Figure S2B). Immunofluorescence of muscle biopsies showed that Clarin‐1 is normally localized to the cell membrane and cytoplasm in healthy muscles (Figure 1O,P). However, in the patient's muscle tissue, the expression of Clarin‐1 was diminished in both locations (Figure 1Q,R). These findings suggest that the c.165C>G and c.407G>A variants of *CLRN1* are pathogenic, as they lead to decreased levels of the Clarin‐1 protein.

### 2.2 Generation of USH3A Patient's iPSCs and 3D Retinal Organoids

We obtained the patient's skin fibroblasts with his informed consent and reprogrammed them into iPSCs. We also derived control iPSCs from a 32‐year‐old male using skin obtained during mole removal surgery. The fibroblasts were cultured from the surrounding non‐pigmented skin and then reprogrammed into iPSCs. Whole‐exome sequencing confirmed that the reprogrammed iPSCs had no genetic mutations.

Both the patient‐derived and control iPSCs were confirmed to have a normal karyotype (Figure S3A) and expressed stem cell markers, including OCT4, SOX2, SSEA4, TRA1‐60, and NANOG (Figure S3B). We generated embryoid bodies from these iPSCs and cultured them with fetal bovine serum to promote differentiation. The differentiated iPSCs expressed AFP, HFN3β, Bruchyury, and PAX6, markers of the endoderm, mesoderm, and ectoderm, respectively, indicating their ability to differentiate into the three germ layers (Figure S3C).

We then assessed Clarin‐1 expression in both the patient‐derived and control iPSCs, as well as in fibroblasts. However, Clarin‐1 was not detected in any of these cell types (Figure S4), highlighting their unsuitability for investigating the mechanisms of USH3A. This underscores the importance of using retinal organoids as models for studying this disease.

**FIGURE 2:**
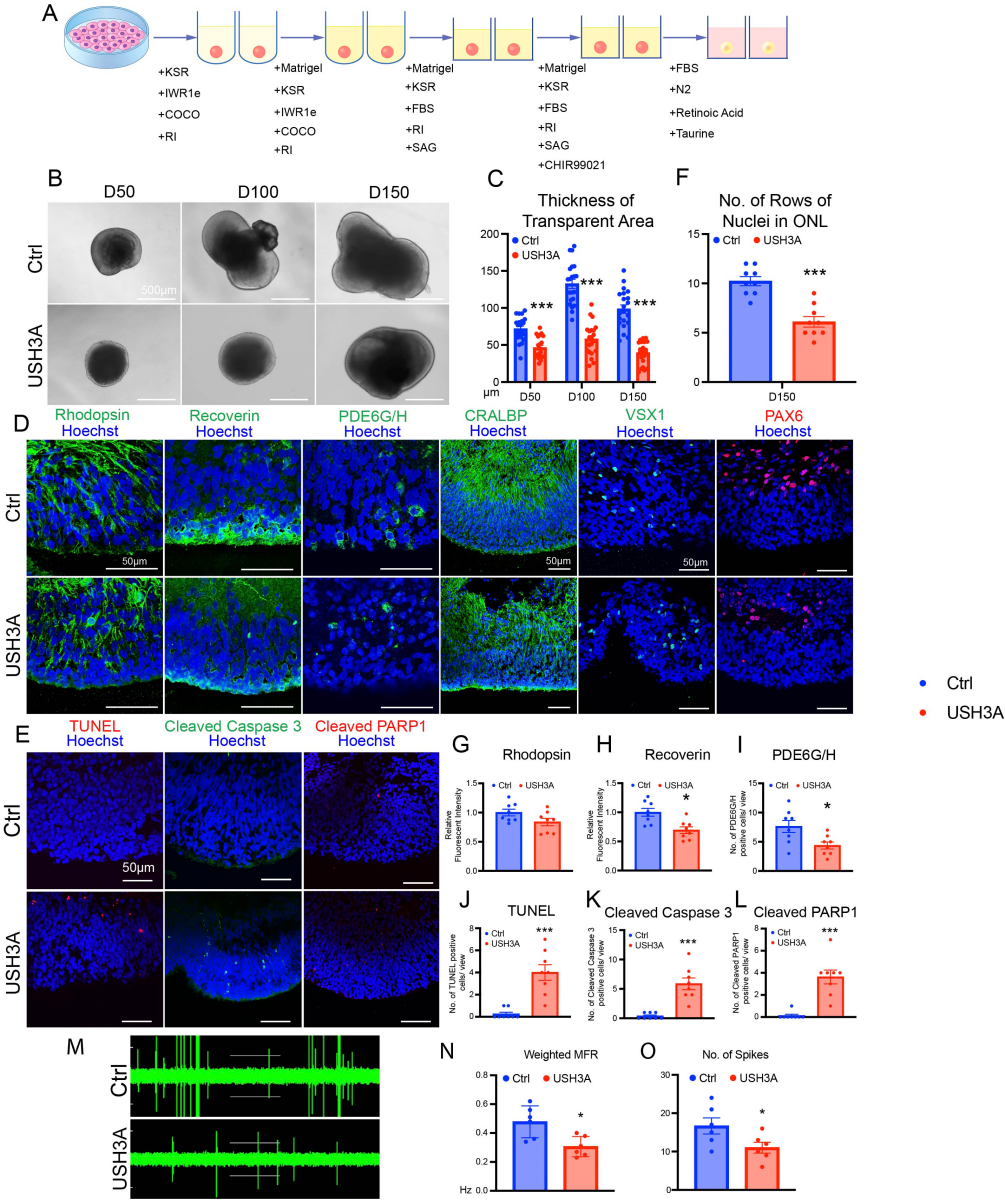
Generation and comparison of retinal organoids from the USH3A patient and control. (A) Protocols for retinal organoids induction and long‐term culturing. (B) Bright‐field images show control and USH3A organoid growth. Scale bar: 500 μm. (C) Quantification of the thickness of the transparent area of the retinal organoids under a microscope. Statistical significance was evaluated by *t*‐test. ***p < 0.001. Data are shown as mean ± SEM. (D) Representative images of immunofluorescence of Rhodopsin, Recoverin, PDE6G/H, CRALBP, VSX1 and PAX6 in control and USH3A retinal organoids. Scale bar: 50 μm. (E) Quantification of the number of rows of nuclei in the ONL. Statistical significance was evaluated by *t*‐test. ***p < 0.001. Data are shown as mean ± SEM. (F–G) Quantification of the relative fluorescent intensity of rhodopsin (F) and recoverin (G) in D. Numbers of samples showed as data points. Statistical significance was evaluated by *t*‐test. *p < 0.05. Data are shown as mean ± SEM. (H) Quantification of the number of PDE6H/G‐positive cells in each field. The number of PDE6H/G‐positive cells was counted across at least 3 randomly selected fields of view per sample. Numbers of samples showed as date points. Statistical significance was evaluated by *t*‐test. *p < 0.05. Data are shown as mean ± SEM. (I–L) Representative images of immunofluorescence of TUNEL staining, cleaved caspase 3 and cleaved PARP1 in control and USH3A retinal organoids(I). Scale bar: 50 μm. Quantification of the number of TUNEL‐ (J), cleaved caspase 3‐ (K) and cleaved PARP1‐ (L) positive cells in each field. The number of TUNEL‐, cleaved caspase 3‐ and cleaved PARP1‐positive cells was counted in at least 3 randomly selected fields of view per sample. Numbers of samples showed as data points. Statistical significance was evaluated by *t*‐test. ***p < 0.001. Data are shown as mean ± SEM. (M–O) Representative raster plots of spontaneous neuronal firing activity in healthy control and USH3A retinal organoids at Day 150 (M). Quantification of the weighted mean firing rate (MFR) (N) and total spike count (O). Numbers of samples showed as data points. Statistical significance was evaluated by *t*‐test. *p < 0.05. Data are shown as mean ± SEM.

We then differentiated the iPSCs into 3D retinal organoids using a published protocol with minor modifications (Figure 2A) [12]. The retinal organoid generated a transparent outer sphere from day 50. Generally, the thickness of those spheres increased until around day 100, after which it slightly decreased by day 150 (Figure 2B,C). However, the thickness of this transparent layer in USH3A retinal organoids was constantly less than that of the normal ones (Figure 2C). Immunofluorescent analysis revealed that the retinal organoids contained multiple layers and expressed markers specific to various retinal cell types (Figure 2D). We measured the number of rows of nuclei in the outer nuclear layer (ONL) and found that the patient's retinal organoids contained fewer nuclei than the control organoids (Figure 2E). Despite this, both control and patient organoids expressed rhodopsin (a rod‐specific marker) and recoverin (a photoreceptor‐specific marker) in the ONL (Figure 2D). Cyclic GMP‐phosphodiesterase 6G/H (PDE6G/H), a marker of cones, was expressed in the ONL of the retinal organoids (Figure 2D). Cellular retinaldehyde‐binding protein (CRALBP, also known as RLBP1), a marker for Müller cells, was expressed around the nucleus in the inner nuclear layer (INL) and extended across the cell body in both control and patient organoids (Figure 2D). VIM, another Müller cell‐specific marker, was highly expressed in the INL and also spread throughout the ONL in the retinal organoids (Figure S5A). Additionally, Visual System Homeobox 1 (VSX1) (Figure 2D) and Paired Box 6 (PAX6) (Figure 2D), markers for bipolar and horizontal cells respectively, were detected in the INL of the retinal organoids.

### 2.3 USH3A Retinal Organoids Exhibit Characteristics of Retinal Degeneration

Quantitative analysis of immunofluorescence intensity revealed a significant reduction in recoverin expression in the USH3A retinal organoids compared with controls, whereas Rhodopsin expression remained relatively unchanged (Figure 2F,G). Furthermore, the number of PDE6G/H‐positive cells was significantly reduced, indicating deficiency in photoreceptor marker expression (Figure 2H). This degeneration was corroborated by an increased apoptotic rate, as evidenced by a higher density of TUNEL‐, cleaved‐caspase 3‐, and cleaved‐poly ADP‐ribose polymerase (PARP) 1‐positive cells in USH3A organoids (Figure 2I–L). Multi‐electrode array (MEA) recordings suggested electrophysiological impairment, with a significantly reduced weighted mean firing rate (MFR) and spike count in USH3A organoids compared with healthy controls (Figure 2M–O). These results suggest that the patient's retinal organoids developed similarly to the controls but exhibited characteristics of retinal degeneration.

### 2.4 Single‐RNA Sequencing of Retinal Organoids

To investigate the transcriptional differences between control and USH3A retinal organoids, we performed single‐cell RNA sequencing on both groups at day 150 using the 10× Genomics platform. For quality control, we first removed cells with fewer than 200 detected genes or more than 6000 detected genes, and those with a mitochondrial gene percentage greater than 10% were also excluded. After extraction and quality control, 16,085 cells from the control organoids and 14,021 cells from the USH3A organoids were analyzed. Sequencing quality was high, with a mean read depth of 39,541 reads per cell in control and 45,727 reads per cell in USH3A organoids, and a sequencing saturation of approximately 72%. The median gene numbers per cell were 2960 and 3247 for control and USH3A organoids, with a median of 6017–6886 UMI counts per cell. In total, more than 35,000 genes were detected across samples.

**FIGURE 3:**
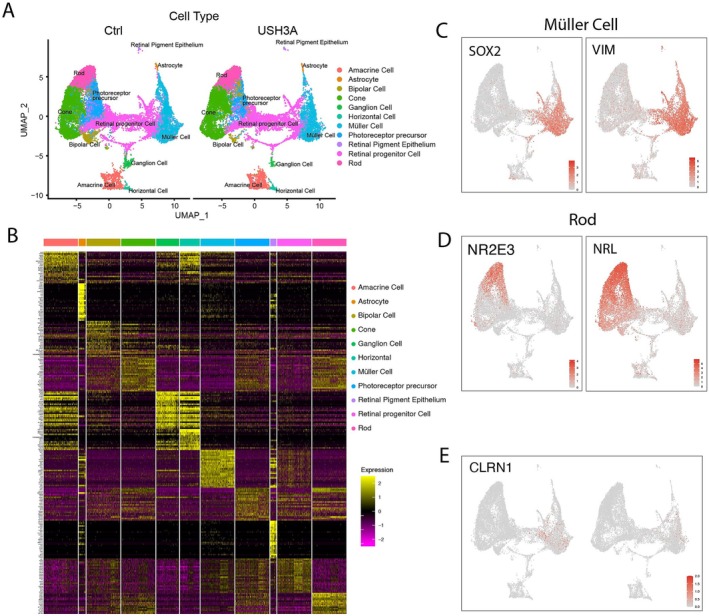
Single‐cell RNA sequencing illustrates cell types in retinal organoids. (A) Clustering of cells from control and USH3A retinal organoids. (B) Heat map showing cluster averages for key genes. (C) UMAP of Müller cell marker *SOX2* and *VIM*. (D) UMAP of rod markers *NR2E3* and *NRL* as indicated. (E) The expression of *CLRN1* in control and USH3A retinal organoids.

Various retinal cell types were identified based on differentially expressed genes (DEGs), including retinal progenitor cells, retinal neurons (rods: NR2E3, NRL; cones: PDE6H, PDE6G; bipolar cells: VSX1, VSX2; ganglion cells: STMN4; amacrine cells: TFAP2A, GAD1; horizontal cells: ONECUT1, ONECUT2), and glial cells including Müller cells (SOX2, VIM) and astrocytes (Figures 3A–D and S6). *CLRN1* was also found in the retinal organoids, suggesting that this human‐derived retinal organoid is an ideal model to study CLRN1‐related retinal degeneration (Figure 3E).

### 2.5 Variants in *CLRN1* Cause Suppression in Genes Related to Visual Function

**FIGURE 4:**
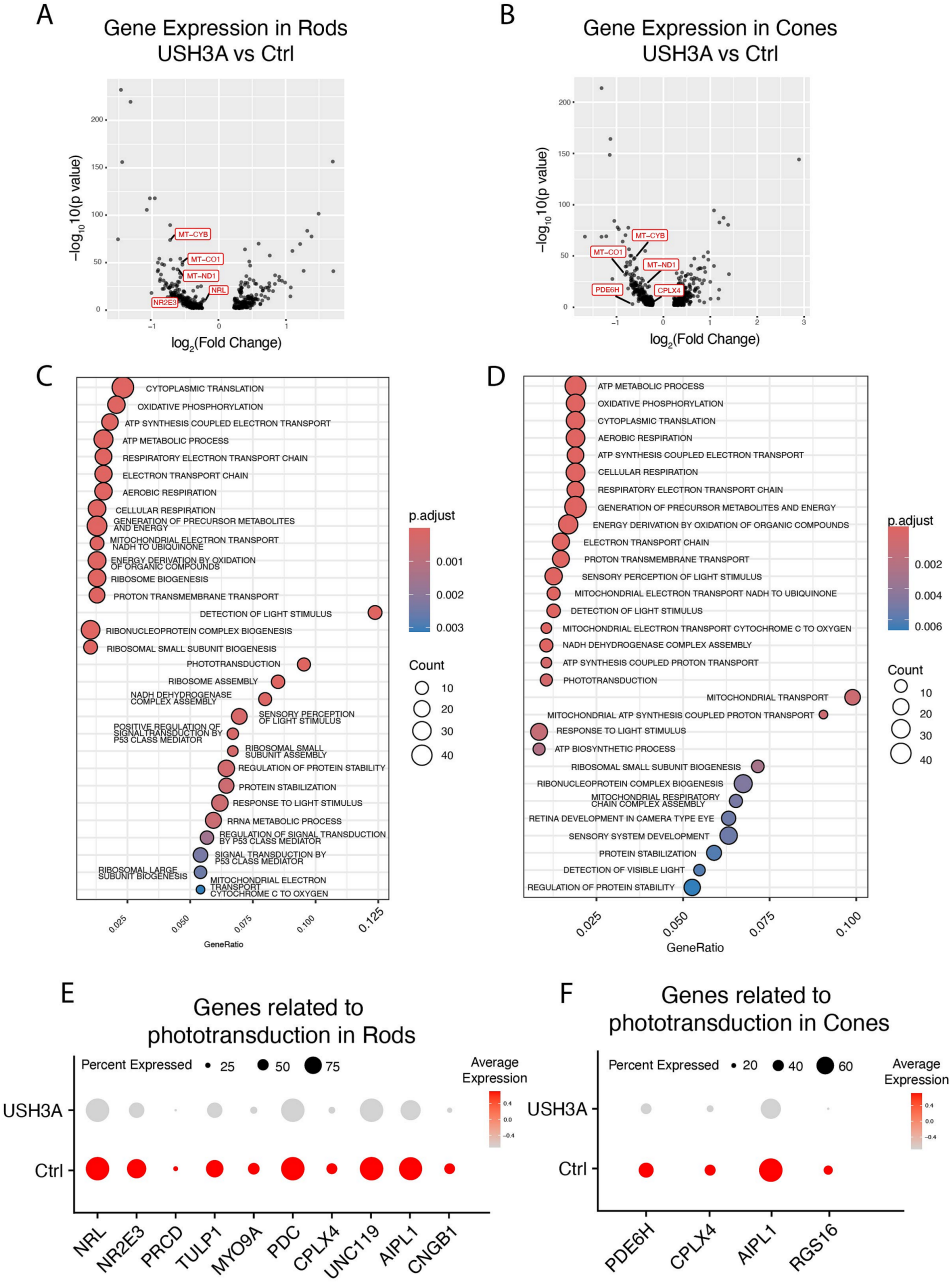
Expression of genes related to visual perception in photoreceptors. (A, B) Volcano plot displaying DEGs between control and USH3A in rods (A) and cones (B). (C, D) Dot plot showing significantly enriched biological processes GO terms on the basis of analysis of DEGs comparing control and USH3A rods (C) and cones (D). (E, F) Bubble plot showing the average expression and percentage of cells expressing genes related to phototransduction in control and USH3A Müller cells.

To explore the impact of *CLRN1* variants on photoreceptor function, we first analyzed the differentially expressed genes (DEGs) in rods and cones from retinal organoids between patient and control and found 200 upregulated genes and 241 downregulated genes in rods, and 260 upregulated genes and 282 downregulated genes in cones (Figure 4A,B). The expression of genes involved in visual perception and phototransduction was significantly decreased in both rods and cones (Figure 4C,D). In rods, 10 phototransduction‐related genes were downregulated, including NR2E3 and NRL (Figure 4E). These two transcription factors are crucial for rod development and the maintenance of photoreceptor function [13]. Mutations in either gene have been linked to retinitis pigmentosa, highlighting their critical role in maintaining healthy photoreceptors [14, 15]. In cones, four genes related to visual function, including PDE6H, were downregulated (Figure 4F). PDE6H encodes the γ subunit of cone‐specific cGMP phosphodiesterase, which plays a key role in visual perception and signal transmission [16]. These findings suggest that *CLRN1* variants impair the expression of genes related to visual function in photoreceptors.

### 2.6 The Expression of Clarin‐1 in the Human Premature Retina and Retinal Organoid

**FIGURE 5:**
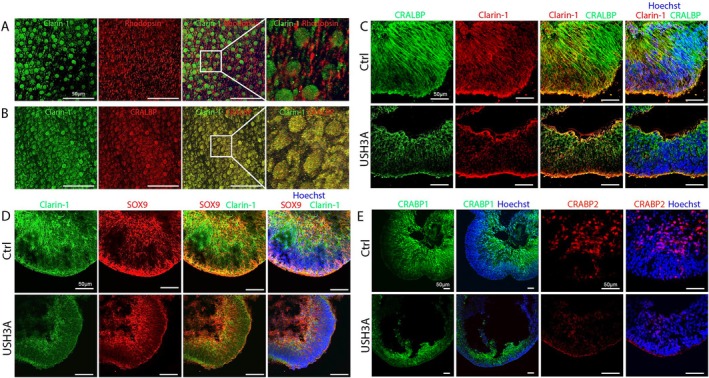
Clarin‐1 expression in human fetal retina and retinal organoids. (A) Clarin‐1 expression in the photoreceptor layer. Rhodopsin served as a photoreceptor marker. Scale bar: 50 μm. (B) Clarin‐1 colocalization with CRALBP, a Müller cell marker. Scale bar: 50 μm. (C) Immunofluorescence illustrating the colocalization of Clarin‐1 and CRALBP in control and USH3A retinal organoids. Scale bar: 50 μm. (D) Immunofluorescence illustrating the colocalization of Clarin‐1 and SOX9 in control and USH3A retinal organoids. Scale bar: 50 μm. (E) Immunofluorescence showing the expression of CRABP1 and CRABP2 in retinal organoids. Scale bar: 50 μm.

To investigate the effect of loss of Clarin‐1 in the retina on photoreceptor degeneration, we first confirmed the compartmental expression of Clarin‐1 in the retina. Previous studies found that Clarin‐1 was expressed in the ribbon synapse of photoreceptors in mouse retina [8, 17], but is specifically expressed in Müller cells in human retina (Figure 5A,B) [8]. To further verify the cell type‐specific expression of Clarin‐1, we collected fetal human retinal tissue and performed immunofluorescence on flat‐mounted retina samples. Our results revealed that Clarin‐1 is not expressed in rhodopsin‐positive rod cells but is localized in cells expressing CRALBP (Figure 5A,B). Moreover, UMAP analysis revealed that *CLRN1* was predominantly expressed in Müller cells, in which VIM and SOX2 were highly expressed, and a small subset of retinal progenitor cells, but not in rods, marked by NR2E3 and NRL (Figure 3C–E). Additionally, immunofluorescence performed on frozen sections of retinal organoids demonstrated similar findings, showing that Clarin‐1 is expressed in CRALBP‐positive cells, consistent with observations in the fetal retina (Figure 5C). The co‐localization of Clarin‐1 with SOX9, a marker of Müller cells and neuroprogenitors, and the partial co‐localization with VIM also suggest the expression of Clarin‐1 in Müller cells in the retinal organoids (Figures 5D and S5B).

### 2.7 Loss of *CLRN1* in Müller Cells Led to Müller Cell Gliosis

Cellular Retinoic Acid‐Binding Protein (CRABP) 1 and 2 are retinoic acid (RA) sensors and essential for Müller cell differentiation and function [18, 19]. CRABP1 combines with retinoic acid to inhibit growth factor sensitivity and promote neural differentiation [20]. CRABP2 can be activated by retinoic acid and promote neurogenesis and attenuate retinal remodeling when the retina is under stress [20, 21, 22]. Next, we analyzed single‐cell RNA data to investigate the differences in expression of the CRABP1 and CRABP2 in Müller cells between the USH3A and control retinal organoids (Figure S7). Bubble diagram analysis illustrated that CRABP1, typically expressed in both control and USH3A Müller cells, exhibited a marked decrease in expression within USH3A Müller cells (Figure S7A). CRABP2, present in approximately 60% of Müller cells, also illustrated a significant reduction in average expression in the USH3A Müller cells (Figure S7A). Immunofluorescence analysis of retinal organoids further validated these findings, indicating reduced levels of CRABP1 and CRABP2 in the INL of the USH3A retinal organoids compared with controls (Figure 5D).

RBP1, a marker of mature Müller cells, is essential for the visual cycle, presenting retinol to generate all‐trans RA, and also regulates photoreceptor structure by influencing outer segment assembly through a rhodopsin‐independent pathway [23]. Our single‐cell analysis indicated that RBP1 was highly expressed in over 90% of both control and USH3A Müller cells, but its average expression was reduced in the USH3A organoids (Figure S7A).

Moreover, single‐cell RNA sequencing and immunofluorescence analysis both identified an upregulation of VIM in the USH3A retinal organoids (Figures S5A,D and S7B). We also observed increased fluorescent intensity of GFAP in the USH3A retinal organoids, although GFAP transcript levels were unchanged (Figures S5C,E and S7B). This is consistent with previous studies that GFAP protein filaments can persist long after the initial transcriptional response has subsided [24, 25, 26]. These findings, together with the downregulation of key homeostatic markers, indicate that CLRN1 variants lead to Müller cell gliosis.

### 2.8 Variants in *CLRN1* Reduced the Expression of Mitochondrial Complex Genes

**FIGURE 6:**
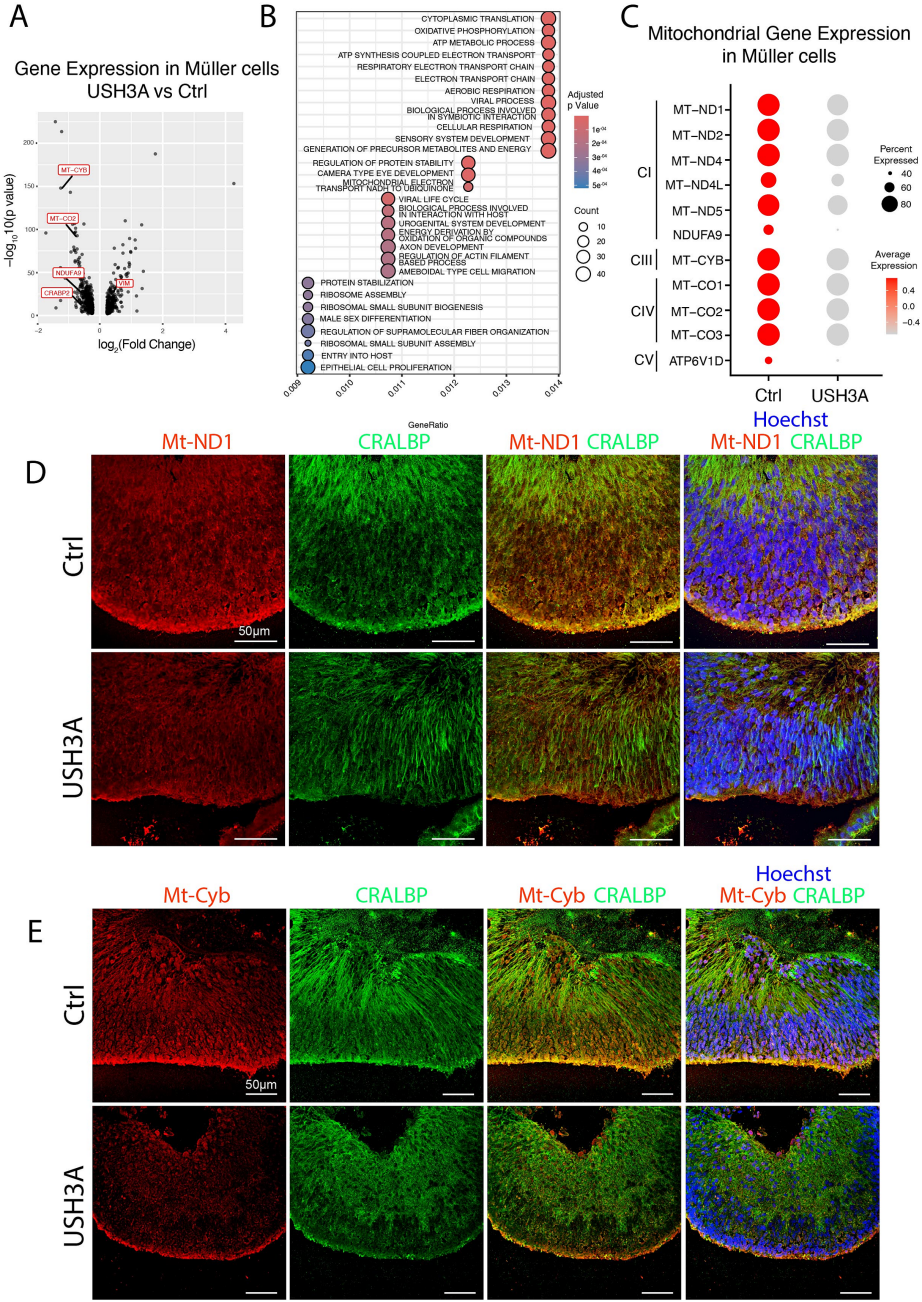
The expression of mitochondrial complex genes in retinal organoids. (A) Volcano plot of DEGs in Müller cells of control and USH3A retinal organoids. (B) Dot plot displaying the significantly enriched GO biological processes terms in Müller cells of control and USH3A retinal organoids. (C) Bubble plot showing the average expression and percentage of cells expressing of mitochondrial genes in control and USH3A Müller cells. CI, CIII, CIV, CV: Mitochondria complex I, III, IV, V. (D) The expression of Mt‐ND1 in control and USH3A retinal organoids. CLALBP was used to stain Müller cells. Scale bar: 50 μm. (E) The expression of Mt‐Cyb in control and USH3A retinal organoids. CLALBP was used to stain Müller cells. Scale bar: 50 μm.

We next performed Gene Ontology (GO) analysis on the DEGs in normal and USH3A Müller cells to investigate the molecular changes occurring in these cells. The expression of 282 DEGs significantly increased and 449 DEGs significantly decreased in USH3A Müller cells (Figure 6A). Notably, nine of the top 15 most significant pathways were associated with mitochondrial oxidative phosphorylation (Figure 6B). In USH3A Müller cells, there was a marked reduction in the expression of multiple genes encoding components of mitochondrial complexes I, III, IV, and V (Figure 6C). This downregulation is likely to cause dysfunction in the electron transport chain, leading to increased levels of reactive oxygen species (ROS), diminished mitochondrial adenosine triphosphate (ATP) production, and reduced membrane potential, factors that are known to contribute to the onset of various diseases [27, 28].

Immunofluorescence analysis further supported these findings, illustrating that Mt‐ND1 (Figure 6D) and Mt‐Cyb (Figure 6E), subunits of mitochondrial complex I and III, respectively, were broadly expressed in retinal organoids. Moreover, these two proteins partially colocalized with CRALBP and their expression was significantly reduced in Müller cells within USH3A retinal organoids. Collectively, these results suggest that loss of Clarin‐1 leads to reduced expression of genes critical to mitochondrial complexes, potentially impairing mitochondrial function in Müller cells.

In addition, pathways related to mitochondria and oxidative phosphorylation were also enriched by GO analysis of DEGs in rods and cones (Figure 4C,D). We observed that 11 mitochondrial genes were downregulated in rods (Figure S8A), and 8 were downregulated in cones (Figure S8B). The expression of genes encoding subunits of Complex I, III, and IV were significantly reduced in rods and cones. This finding suggests that *CLRN1* variants adversely affect mitochondrial function in photoreceptors as well.

### 2.9 Variants in *CLRN1* Caused Mitochondrial Dysfunction MIO‐M1 Cells

**FIGURE 7:**
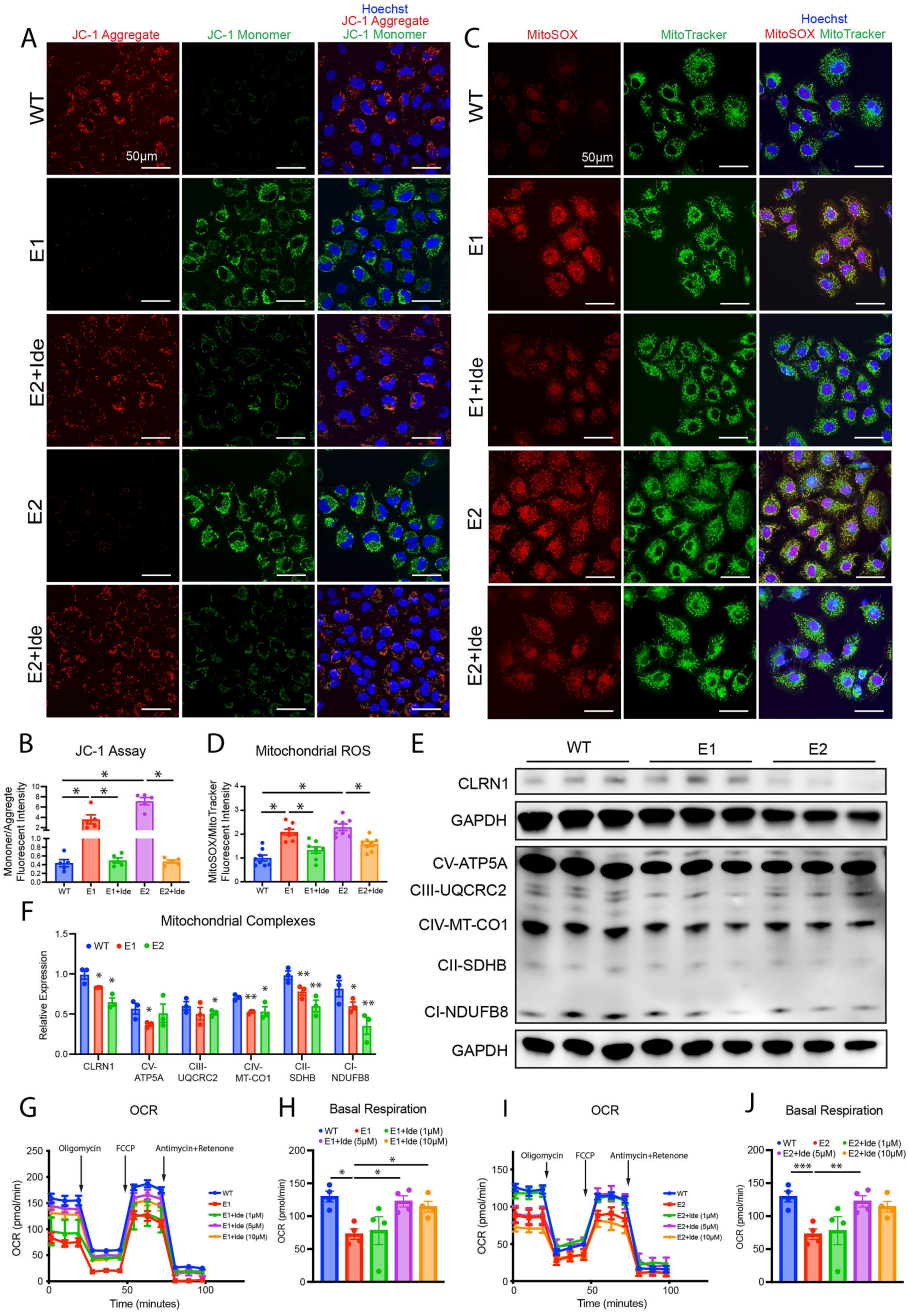
Effects of variants in CLRN1 on mitochondrial function. (A, B) JC‐1 assay of MIO‐M1 cells transfected with WT, E1 and E2 (A). 5 μM Idebenone was applied for 12 h before screening. Scale bar: 50 μm. Quantification of the fluorescence intensity ratio of JC‐1 monomer to JC‐1 aggregates (B). Statistical significance was evaluated by *t*‐test. *p < 0.05. Data are shown as mean ± SEM. (C, D) MitoSOX and MitoTracker staining of MIO‐M1 cells transfected with WT, E1 and E2 (C). 5 μM Idebenone was applied for 12 h before screening. Scale bar: 50 μm. Quantification of the fluorescence intensity ratio of MitoSOX‐stained area to MitoTracker‐stained area (D). Statistical significance was evaluated by *t*‐test. *p < 0.05. Data are shown as mean ± SEM. (E, F) Western blot of mitochondrial complexes in MIO‐M1 cells transfected with WT, E1 and E2 (E). CI, CII, CIII, CIV, CV: Mitochondria complex I, II, III, IV, V. Quantification of mitochondrial complexes expression (F). Numbers of samples shown as data points. Statistical significance was evaluated by *t*‐test. *p < 0.05, **p < 0.01. Data are shown as mean ± SEM. G‐J. Respiration assays. OCR of MIO‐M1 cells transfected with WT, E1 and E2, treated with 1, 5, 10 μM Idebenone at each time point (G, I). Oligomycin, FCCP, rotenone and antimycin A were applied at the indicated time points to study mitochondrial functions. Basal respiration of MIO‐M1 cells transfected with WT, E1 and E2, treated with 1, 5, 10 μM Idebenone (H, J). Statistical significance was evaluated by one‐way ANOVA followed by Dunnett's multiple comparisons test. *p < 0.05, **p < 0.01, ***p < 0.001. Data are shown as mean ± SEM.

To investigate how *CLRN1* variants affect mitochondrial function in Müller cells, we transfected the human Müller cell line MIO‐M1 with either wild‐type (WT), E1, or E2 *CLRN1* variants. We assessed the mitochondrial membrane potential, a critical factor for proper mitochondrial function, using JC‐1 staining (Figure 7A,B). In cells transfected with WT *CLRN1*, the JC‐1 dye formed red aggregates, indicating healthy mitochondrial membrane potential. However, in the cells transfected with the E1 or E2 variants, the dye shifted to green, signifying a reduced membrane potential. This reduction in membrane potential was partially restored by treating the cells with 5 μM idebenone, a ubiquinone analog used to treat mitochondrial disorders such as Leber's hereditary optic neuropathy (LHON). This suggests that the mitochondrial dysfunction caused by the E1 and E2 variants is partially reversible [29]. We also evaluated the levels of ROS using MitoSOX staining, with MitoTracker used to visualize mitochondria (Figure 7C,D). Consistent with the JC‐1 results, MIO‐M1 cells transfected with E1 and E2 variants exhibited approximately twice the ROS levels compared to WT‐transfected cells, and idebenone treatment was able to partially reduce ROS production.

Western blot analysis was performed to study changes in the expression of mitochondrial complexes in the transfected MIO‐M1 cells (Figure 7E,F). The expression levels of *CLRN1* were approximately 15% lower in cells with the E1 variant and 40% lower in cells with the E2 variant compared to WT. Furthermore, the expression of subunits from all five mitochondrial complexes was significantly reduced in cells with E1 and E2 variants.

The oxidative phosphorylation function was assessed using the Agilent Seahorse XF Assay. The oxygen consumption rate (OCR) was measured before and after the addition of Oligomycin (a complex V inhibitor), carbonyl cyanide 4‐(trifluoromethoxy) phenylhydrazone (FCCP, an uncoupler of mitochondrial oxidative phosphorylation), antimycin A (a complex III inhibitor), and rotenone (a complex I inhibitor). The OCR was lower in the cells with the E1 or E2 variant compared to WT (Figure 7G,I). The basal respiration, maximal respiration, and ATP production were significantly reduced in E1 and E2‐transfected cells compared with cells transfected with WT (Figures 7H,J and S9A–D). Interestingly, treatment with idebenone at different concentrations could partially improve OCR basal respiration, maximal respiration, and ATP production in the cells transfected with E1 and E2, and that 5 μM idebenone had a better effect than other concentrations (Figures 7G–J and S9A–D).

Together these findings indicate that *CLRN1* variants reduce the expression of mitochondrial complex genes, leading to mitochondrial dysfunction that is, to some extent, reversible with idebenone treatment.

### 2.10 *CLRN1* Variants in MIO‐M1 Cells Induced Time‐Dependent Impairment in 661W Cells

**FIGURE 8:**
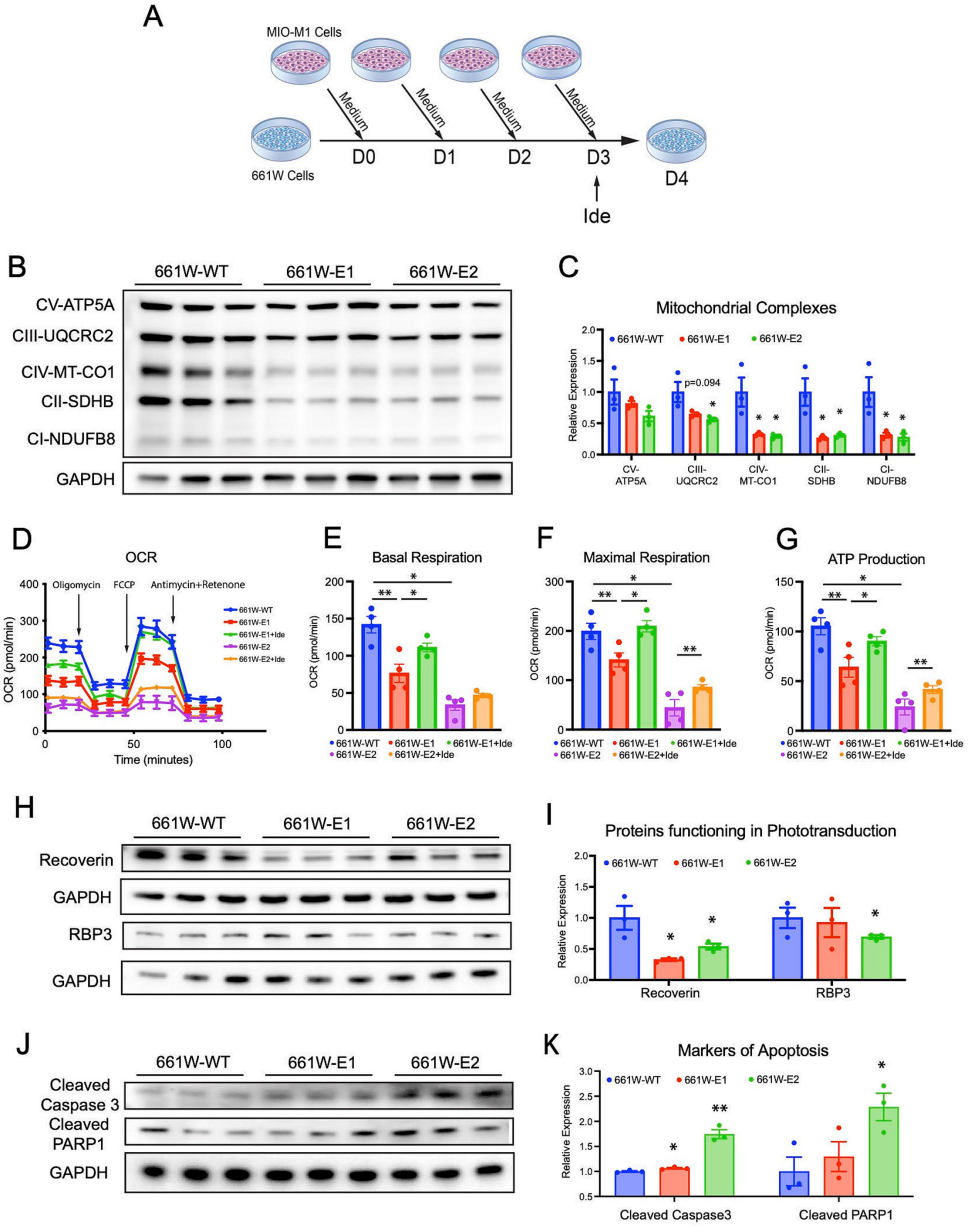
Impact of CLRN1 variants in MIO‐M1 cells on mitochondria, protein expression, and survival of 661W cells after 4‐day treatment. (A) Schematic representation of the conditioned medium transfer experiment. MIO‐M1 Müller cells were transfected with WT or CLRN1 variants (E1, E2) for 48 h, followed by medium collection. This medium was then applied to 661W photoreceptor‐like cells, with daily medium refreshment for 4 days. The 5 μM Idebenone was added 24 h before further experiments. (B, C) Western blot of mitochondrial complexes of 661W cells treated with medium of MIO‐M1 cells transfected with WT, E1 and E2 for 4 days (B). CI, CII, CIII, CIV, CV: Mitochondria complex I, II, III, IV, V. Quantification of mitochondrial complex expression (C). Numbers of samples showed as data points. Statistical significance was evaluated by *t*‐test. *p < 0.05. Data are shown as mean ± SEM. (D–G) Respiration assays. OCR of 661W cells treated with medium from MIO‐M1 cells transfected with WT, E1 and E2, treated with 5 μM idebenone on Day 4 (D). Oligomycin, FCCP, rotenone and antimycin A were applied at indicated time points to study mitochondrial functions. Basal respiration (E), maximal respiration (F), and ATP production (G) of 661W cells treated with medium from MIO‐M1 cells transfected with WT, E1 and E2 treated with 5 μM Idebenone on Day 4. Numbers of samples showed as data points. Statistical significance was evaluated by *t*‐test. *p < 0.05, **p < 0.01. Data are shown as mean ± SEM. (H, I) Western blot analysis of photoreceptor markers, Recoverin and RBP3, in 661W cells after 4 days treated with medium from MIO‐M1 cells (H). Quantification of protein expression (I). Numbers of samples showed as data points. Statistical significance was evaluated by *t*‐test. *p < 0.05. Data are shown as mean ± SEM. (J, K) Evaluation of apoptosis markers, cleaved caspase‐3 and cleaved PARP‐1, in 661W cells treated with medium from MIO‐M1 cells containing WT, E1 or E2 for 2 days. (J) Quantification of protein expression (K). Numbers of samples showed as data points. Statistical significance was evaluated by *t*‐test. *p < 0.05, **p < 0.01. Data are shown as mean ± SEM.

To investigate whether *CLRN1* variants in Müller cells affect photoreceptor health, we treated 661W photoreceptor cells with medium collected from MIO‐M1 cells transfected with either wild‐type, E1, or E2 *CLRN1* variants (hereafter referred to as 661W‐WT, E1, E2) and refreshed the medium daily. We monitored the cellular response of 661W cells at 2 and 4 days post‐treatment (Figures 8A and S10A).

The expression of mitochondrial complexes in 661W cells was decreased after treatment. The expression of Mt‐CO1 and NDUFB8 was reduced by ~50% in 661W cells treated with medium from MIO‐M1 cells transfected with E1 and E2, compared with those transfected with WT on Day 2 (Figure S10B,C). By Day 4, this impairment became more extensive. Western blot of UQCRC2, Mt‐CO1, SDHB, and NDUFB8 illustrated a more profound downregulation, with protein levels decreasing by 40%–80% in 661W‐E1/E2 compared with 661W‐WT (Figure 8B,C).

This structural impairment was accompanied by significant functional deficits. Bioenergetic analysis using Seahorse assays revealed that 661W‐E1/E2 cells exhibited significantly reduced basal respiration, maximal respiration, and ATP production compared with 661W‐WT (Figure 8D–G). Consistent with these findings, 661W‐E1/E2 cells displayed reduced mitochondrial membrane potentials and elevated ROS levels (Figure S11). Importantly, a 24‐h treatment with 5 μM idebenone on Day 4 partially rescued these metabolic deficits, including the restoration of mitochondrial membrane potential and mitigation of oxidative stress (Figures 8A,D–G and S11). These results demonstrate that *CLRN1* variants in Müller cells induce a progressive and partially reversible mitochondrial impairment in photoreceptor cells.

The protein levels of recoverin and Retinol‐Binding Protein (RBP) 3, pan‐photoreceptor markers expressed in both rods and cones, stayed unchanged on Day 2 but decreased on Day 4 in 661W cells treated with medium from MIO‐M1 cells with E1 and E2 (Figures 8H,I, and S10D,E). Similarly, the expression of apoptosis markers, cleaved caspase 3 and cleaved PARP1, was relatively stable in 661W‐E1/E2 cells on Day 2 but markedly elevated by Day 4 (Figures 8J,K and S10F,G). Collectively, these findings indicate that Müller cells with variants in *CLRN1* exert a time‐dependent cytotoxic effect, which progressively impairs photoreceptor identity and triggers apoptotic cell death. This cascade of events suggests that Müller cell dysfunction serves as a critical driver in the degeneration of photoreceptors associated with *CLRN1* variants.

## 3 Discussion

*CLRN1*, which encodes Clarin‐1, is responsible for USH3A, an autosomal recessive disorder marked by progressive sensorineural deafness, retinitis pigmentosa, and variable vestibular dysfunction. Here, we report a patient with two heterozygous variants, c.165C>G (p.D55E) and c.407G>A (p.G136E), in *CLRN1*. Our findings reveal that both variants reduce Clarin‐1 expression. To explore the mechanism by which *CLRN1* leads to retinitis pigmentosa, we collected the patient's fibroblasts, produced iPSCs, and generated retinal organoids. *CLRN1* was found to be specifically expressed in Müller cells. Loss of *CLRN1* compromises Müller cell identity, evidenced by the downregulation of cell‐specific markers, and disrupts mitochondrial bioenergetics. These changes in Müller cells propagate to photoreceptors, leading to a progressive loss of photoreceptor markers, increased apoptosis, and diminished electrophysiological function. Our results suggest that the c.165C>G (p.D55E) and c.407G>A (p.G136E) variants in *CLRN1* are pathogenic mutations and that mitochondrial dysfunction in Müller cells contributes to retinitis pigmentosa.

In mice, *CLRN1* expression is detectable in the retina at postnatal Day 7 (P7) but diminishes and becomes undetectable by Week 4 and into adulthood. In contrast, *CLRN1* is continuously expressed in the human retina and is specifically localized to Müller cells, not in other cell types like rods or cones. Therefore, generating patient‐derived USH3A retinal organoids is essential for investigating the molecular mechanisms of USH3A, as it more accurately reflects human retinal biology. We employed a protocol whereby the retinal organoids develop from a 3D structure, with photoreceptors appearing as early as Week 3, and all cell types persist for an extended period [30, 31, 32]. This protocol mimics retinal development, and its ability to produce various cell types simultaneously makes it an effective model for studying intercellular relationships. Our results demonstrated that the USH3A retinal organoids had reduced expression of phototransduction‐related genes in both rods and cones, with rods being more affected. This finding aligns with the patient's clinical manifestations and previous studies, that USH3A patients experience retinitis pigmentosa and uncorrectable blurred vision, implicating cone involvement. It is also consistent with previous studies that mutations in MYO7A, the disease‐causing gene of USH1B, disturbed the expression of genes in visual perception and phototransduction in both rods and cones of USH1B retinal organoids [33]. However, this study generating retinal organoids for USH1B, one of the most severe types of USH, did not observe obvious retinal degenerative features [33]. Researchers in these studies used a 2D‐based protocol, where retinal organoids transitioned through a 2D stage, with photoreceptors appearing around Week 24 [34, 35]. Consequently, the photoreceptors existed for only a few weeks, even though the retinal organoids were cultured for 180 days. These photoreceptors may not have been “aged” enough to display degenerative characteristics.

Our study is the first to clarify the pathogenicity of the c.165C>G and c.407G>A variants. While the c.407G>A variant was previously reported, its pathogenic mechanism had not been verified. The c.165C>G variant is a novel mutation. Our results indicate that either variant could reduce the *CLRN1* mRNA levels and protein expression. In addition to these two missense mutations, previous studies have reported multiple mutations in *CLRN1*, including deletion mutations c.459_461delATT [36] and c.341delT:p.V114Gfs*22 [37]; deep intronic mutation c.254–649T >G [38]; nonsense mutation p.Y63X stop mutation [37]; and missense mutations c.161T>C (p.L54P) [39], c.118T>G (p.C40G) [40], c.449T>C (p.L150P) and c.144T>G (p.N48K) [41], c.118T>G (p.C40G) [2], c.313T>C (p.S105P) [42], c. T300G (p.Y100X) and c. T131A (p.M44K) [36], c.C619T (p.R207X) and c.T503A (p.I168N) [43] and c.92C>T (p.P31L) and c.461T>G (p.L154W) [44]. The mechanisms by which these mutations cause USH3A vary, but most lead to reduced Clarin‐1 protein levels, indicating that loss of Clarin‐1 is a primary cause of USH3A. For example, the p.N48K mutation, one of the most common missense mutations, abolishes N‐linked glycosylation, reduces Clarin‐1 at the cell membrane while increasing its aggregation in the endoplasmic reticulum, and decreases Clarin‐1 stability [7, 39, 45]. The deep intronic mutation c.254–649T >G in *CLRN1* has been reported to cause USH3A by creating a novel donor splice site, resulting in a frameshift and premature termination codon, leading to an incomplete Clarin‐1 protein [38].

Our results indicate that *CLRN1* is a Müller cell‐specific gene, supported by the colocalization of *CLRN1* and CRALBP in retinal organoids and fetal retina. Müller cells are retinal‐specific glial cells that span the entire thickness of the retina, supporting neurons in various ways. Our results illustrate that loss of *CLRN1* in Müller cells leads to gliosis, a hallmark of retinal stress consistent with many models of retinitis pigmentosa [46]. Malfunctioning Müller cells not only disrupt homeostatic support through known mechanisms like cytokine secretion [47], microglial activation [48], and ion dysregulation [49], but are also intrinsically linked to mitochondrial dysfunction [50], which serves as a central driver of this metabolic and functional collapse.

Our results illustrate that loss of *CLRN1* suppresses the expression of mitochondrial complex subunits and induces mitochondrial dysfunction in Müller cells. Mitochondria act as essential bioenergetic hubs that fuel the neuroprotective functions of Müller cells, while also strictly regulating ROS production, ion homeostasis, and cell survival [51, 52]. Given that mitochondrial dysfunction typically impairs these neuroprotective capacities, often triggering Müller cell gliosis and secondary retinal degeneration [53], we hypothesized that the mitochondrial defects observed in our USH3A model are central to the underlying pathology.

We performed conditioned medium experiments to investigate the impact of Müller cell impairment on photoreceptor health. Our cell‐based results are highly consistent with the phenotypes observed in *CLRN1*‐mutant retinal organoids. Specifically, conditioned medium from MIO‐M1 cells with *CLRN1* variants triggered a mitochondrial impairment in 661W cells that mirrored the mitochondrial complex downregulation observed in our organoid photoreceptors. Furthermore, the downstream consequences, including reduced metabolic function, elevated oxidative stress, and eventual apoptosis, align with the functional decline and the increased cell death noted in our organoid models. These results indicate that *CLRN1* variants in Müller cells likely induce photoreceptor degeneration through a paracrine‐mediated mechanism. These findings are consistent with previous studies that Müller cells function as metabolic and structural supporters of photoreceptors [54, 55]. Notably, mitochondrial impairment in 661W cells precedes the loss of photoreceptor markers and the induction of apoptosis, suggesting that mitochondrial dysfunction may act as a primary insult rather than a secondary consequence of cell death. This temporal hierarchy highlights the link between Müller cells and photoreceptors, confirming that this mitochondrial axis is a key target for therapeutic intervention. The precise molecular mediators of this crosstalk remain to be fully characterized in future studies.

Our results suggest that idebenone, a mitochondrial‐targeted therapy, emerges as a potential treatment of retinal degenerative diseases. Previous studies have shown its therapeutic benefits in LHON, improving best‐corrected visual acuity and color vision [56, 57]. Research also demonstrates that idebenone reduces ROS levels in photoreceptor cells in retinal detachment models, improves retinal function in retinal dystrophy models, and protects mitochondria from oxidative damage [58, 59]. Our findings further support idebenone's potential in retinal diseases by ameliorating mitochondrial function in Müller cells. These collective results suggest that idebenone could be a promising avenue for treating retinal degenerative diseases, including USH3A.

One limitation of this study is that it was conducted using fibroblasts from a single patient, which restricts the generalizability of the findings. Given the diversity of *CLRN1* mutations across different patients, phenotypic outcomes may vary, and larger studies involving a more diverse cohort are needed to validate these results. Additionally, while the retinal organoids derived from iPSCs offer a valuable model for studying retinal degeneration, they cannot fully replicate the complexity of the human retina in vivo. Moreover, although we identified that variants in *CLRN1* lead to mitochondrial dysfunction, the underlying mechanisms remain unclear. Further investigations into the relationship between Clarin‐1 and mitochondrial function are necessary to elucidate this connection.

In summary, we found that Clarin‐1 is specifically expressed in Müller cells within the human retina and retinal organoids. Variants in *CLRN1* impaired mitochondrial function in Müller cells, suppressed the expression of Müller cell‐specific markers, and led to photoreceptor degeneration. These findings provide further evidence that Müller cell dysfunction plays a crucial role in retinal degeneration, suggesting that targeting Müller cells could offer new therapeutic strategies for treating retinal degenerative diseases.

## 4 Methods

### 4.1 Fibroblast Culturing and iPSC Reprogramming

Skin tissues were collected from consenting patients undergoing muscle biopsy. The skin samples were finely chopped and cultured in DMEM supplemented with 10% fetal bovine serum (FBS). Fibroblasts were transfected via electroporation using a 2D Nucleofector system (LONZA, AAB‐1001) and a primary cell Nucleofector Kit (LONZA, VVPI‐1005) with plasmids encoding SOX2, KLF4, L‐MYC, LIN28, OCT3/4, and shRNA against p53. The transfected cells were seeded onto a plate coated with 1% Matrigel and initially cultured in DMEM with 10% FBS. The medium was replaced with ReproTeSR Medium (2‐Component) (StemCell Technologies, 05926) the following day, with subsequent media changes every 2 days. Skin from a normal control, obtained during a mole removal surgery, was used to derive fibroblasts and reprogrammed into iPSCs, as previously described.

Embryonic stem cell‐like colonies were manually selected, transferred to a new 1% Matrigel‐coated plate, and cultured in mTeSR Plus medium. The induced pluripotent stem cells (iPSCs) were passaged every 7–10 days using DBPS containing 500 μM EDTA. For long‐term culture, the iPSCs were maintained in mTeSR Plus, supplemented with 10 μM Y‐27632 for the first day following passaging, and then continued in mTeSR Plus.

### 4.2 Retinal Organoid Culturing

iPSCs were harvested, and 9000–12,000 cells were seeded into each well of a low‐adherent U‐bottom 96‐well plate with 100 μL of Medium I, consisting of Glasgow's Eagle's Minimal Essential Medium (GMEM) supplemented with 20% knockout serum replacement (KSR), 100 μM non‐essential amino acids (NEAA), 1 mM pyruvate, 3 μM IWR‐1e, 20 μM Y‐27632, 30 ng/mL COCO, and 100 μM β‐mercaptoethanol (β‐ME). On Day 2, each well was topped up with 100 μL of Medium I containing 2% growth factor‐reduced (GFR) Matrigel. On Days 6 and 9, half of the medium was replaced with 100 μL of Medium I containing 1% GFR Matrigel.

On Day 12, the cell pellets were transferred to a low‐adherent 24‐well plate containing Medium II, which comprised GMEM, 20% KSR, 10% FBS, 1% GFR Matrigel, 100 μM NEAA, 1 mM pyruvate, 100 nM potent smoothened receptor agonist (SAG), 20 μM Y‐27632, and 100 μM β‐ME. On Day 15, the medium was replaced with Medium II containing 3 μM CHIR99021. The medium was subsequently changed to Neural Retina Culture Medium, consisting of DMEM/F12‐Glutamax, 10% FBS, 100 μM NEAA, 1 mM pyruvate, N2 supplement, 500 nM retinoic acid, and 100 μM taurine on Day 18. Pigmented tissue was removed under the microscope using needles. The Neural Retina Culture Medium was changed twice per week for long‐term culturing.

### 4.3 Embryoid Body Formation

iPSCs were harvested, and approximately 20,000 cells in 20 μL of mTeSR Plus medium containing 10 μM Y‐27632 were seeded onto the lid of a low‐adherent Petri dish and left to suspend overnight. The resulting cell clumps were collected and cultured in DMEM/F12 supplemented with 10% FBS, 100 μM NEAA, 1 mM pyruvate, and 100 μM β‐ME for 3 days. Subsequently, the embryoid bodies were transferred onto coverslips coated with 1% Matrigel and cultured for 2 weeks. Afterward, the embryoid bodies were fixed and subjected to immunofluorescence staining for identification.

### 4.4 Cryosection, Immunofluorescent Staining, Thickness Measurement and Retinal Cell Quantification

Retinal organoids were fixed with 4% paraformaldehyde (PFA) and embedded in OCT compound. Retinal organoids were sectioned into 10–12 μm slices using a Leica cryostat. For Immunofluorescent staining, the slides or cells were blocked with PBS containing 10% normal donkey serum for 1 h, followed by overnight incubation with primary antibodies at 4°C. The following day, the slides were incubated with secondary antibodies for 2 h and stained with Hoechst for 10 min at room temperature. For TUNEL staining, we employed the Beyotime C1089 One‐Step TUNEL Apoptosis Assay Kit according to the manufacturer's instructions. Images were captured using an Olympus confocal microscope. Detailed antibody information is provided in Table S1.

The thickness of the transparent area in retinal organoids was measured in four representative regions of each organoid. For each group, 8–10 retinal organoids were measured. The number of nuclear rows in the ONL was quantified in three representative regions of each image and averaged to determine the mean number of nuclei per retinal organoid. Fluorescence intensity was quantified using ImageJ software by outlining the regions of interest (ROI) to calculate the mean integrated density. For cell quantification, including PDE6G/H‐positive cells and apoptotic markers (TUNEL, cleaved‐caspase 3, and cleaved‐PARP1), positive cells were counted in randomly selected representative fields of view (3 fields per organoid). Data are presented as the mean number of positive cells per field or mean fluorescence intensity. A total of 8–10 retinal organoids were assessed for each group.

### 4.5 MEA Recording and Data Analysis

Retinal organoids (Day 150) were transferred to GFR Matrigel‐coated MEA plates recording to ensure stable tissue‐electrode contact. Electrophysiological activity was recorded for 10 min per organoid using an Axion Biosystems MEA system.

Raw data were processed using the AxIS Navigator software (version 3.9.1). Spikes were detected using an adaptive thresholding method set at 6 times the standard deviation of the root‐mean‐square (RMS) noise. The weighted MFR was calculated based on the activity of active electrodes (defined as those with > 0.1 spikes/s). Data were analyzed to compare the overall neuronal excitability and spike frequency between healthy control and USH3A organoids.

### 4.6 Single‐Cell RNA Sequencing

Retinal organoids were harvested on Day 150 and digested in a solution containing 2 mg/mL collagenase at 37°C for 30 min. The resulting cell suspension was passed through a 40 μm cell strainer and centrifuged at 300 rcf at 4°C for 5 min to remove debris. The cell pellet was resuspended in PBS containing 0.04% bovine serum albumin, and the cells were counted using trypan blue exclusion on a Countess II automated cell counter.

cDNA libraries were prepared using the Chromium Single Cell 3′ Library & Gel Bead Kit v2 (10× Genomics) and sequenced on an Illumina NovaSeq 6000 PE150. Sequence data were converted to FASTQ format using Illumina bcl2fastq software. The 10× Genomics Cell Ranger Single‐Cell Software Suite v7.0.0 was employed for sample demultiplexing, barcode processing, single‐cell 3′ gene counting, and mapping to the Human Reference Genome GRCh38‐2020‐A. Differential expression analysis was performed using the limma‐voom method with a cut‐off of adjusted *p*‐value < 0.05 for statistical significance. GO analysis was conducted using the clusterProfiler package (version 3.0.4).

### 4.7 Cell Culture and Transfection

MIO‐M1 and HEK‐293T cells were cultured in DMEM with 10% FBS and passaged using trypsin. Cells were seeded into 6‐well plates. For transfection, the cells were incubated in Opti‐MEM containing 4 μL of Lipofectamine 2000 and 2.5 μg of plasmid DNA for 6 h. After this incubation period, the medium was replaced with DMEM containing 10% FBS. Cells were harvested 24–48 h post‐transfection for further analysis.

For conditioned medium experiments, MIO‐M1 cells were transfected with WT or *CLRN1* variants (E1, E2) and cultured for 48 h. The conditioned medium was then collected, centrifuged to remove cell debris, and applied to 661W cells seeded in 6‐well plates. The 661W cells were treated with this medium, refreshed daily, for a total duration of 2 or 4 days prior to downstream biochemical and functional analysis.

### 4.8 Western Blot

Proteins were extracted using RIPA buffer supplemented with 1% Protease and Phosphatase Inhibitor Cocktail. The concentration of the extracted proteins was determined using the Micro BCA Protein Assay Kit. A total of 20 μg of protein was separated by electrophoresis on 4%–12% bis‐Tris SDS‐polyacrylamide gels and subsequently transferred onto a polyvinylidene fluoride (PVDF) membrane. The membrane was incubated with primary antibodies overnight, followed by incubation with secondary antibodies for 2 h. Protein signals were detected using the ECL Western Blotting System and analyzed with ImageJ software. Details regarding the antibodies are provided in Table S1. The unedited full‐length blots and gels for this assay are provided in Data S1.

### 4.9 OCR Measurement

The OCR was measured with a Seahorse XF96 extracellular flux analyzer. MIO‐M1 or 661W cells (1 × 10^4^–2 × 10^4^) were seeded in each well of a Seahorse XF96 polystyrene tissue culture plate. The following compounds were sequentially added at specified time points: oligomycin (2 μM), FCCP (3 μM), antimycin A (2 μM), and rotenone (2 μM).

The basal OCR was calculated by subtracting the OCR after rotenone/antimycin A treatment from the OCR before oligomycin addition. Maximal OCR was determined by subtracting the non‐mitochondrial OCR from the OCR after FCCP treatment. ATP production was assessed by subtracting the OCR after oligomycin treatment from the OCR before oligomycin treatment.

### 4.10 JC‐1 and MitoSOX Staining

Mitochondrial membrane potential was assessed using the JC‐1 Mitochondrial Membrane Potential Assay Kit. Cells were incubated in DMEM containing JC‐1 dye for 20 min, followed by Hoechst staining for 10 min. Fluorescent signals were immediately captured using an Olympus confocal microscope.

Mitochondrial ROS generation was measured using MitoSOX staining, with mitochondria visualized by MitoTracker. Cells were incubated in DMEM containing 0.5 μM MitoSOX for 20 min and 500 nM MitoTracker for 20 min, followed by Hoechst staining. Images were taken immediately using an Olympus confocal microscope.

### 4.11 Statistics

Statistical analysis was performed using Prism 8.0 or Microsoft Excel. The number of replicates for each experiment is indicated in the figure legends. Error bars in the graphs represent means ± SEM. Normality of the data was assessed using the Shapiro–Wilk test, and all datasets were found to follow a normal distribution. Statistical significance was evaluated using a two‐tailed Student's *t*‐test for pairwise comparisons or one‐way ANOVA followed by Dunnett's multiple comparisons test for dose–response experiments, and differences were considered significant at *p* < 0.05.

## Funding

This work was supported by National Natural Science Foundation of China, 82201169, 32270715, 82471090; China Postdoctoral Science Foundation, 2022M711952, 2022TQ0198; Natural Science Foundation of Shandong Province, 2601010520249H; Qilu Young Scholar Program of Shandong University, 20201125.

## Ethics Statement

Human sample collection and related procedures were approved by the Medical Ethics Committee of Qilu Hospital of Shandong University (Approval ID: 2020067). All participants provided written informed consent before inclusion in the study. All research was conducted in accordance with the Declaration of Helsinki and institutional ethical guidelines.

## Conflicts of Interest

The authors declare no conflicts of interest.

## Supporting information


**Figure S1:** Conservation analysis of the *CLRN1* variants.
**Figure S2:** mRNA levels of CLRN1 in muscles and HEK293 cells.
**Figure S3:** Characterization of iPSCs for the patients.
**Figure S4:** Clarin‐1 expression in fibroblasts and iPSC.
**Figure S5:** The expression of Vimentin (VIM) in retinal organoids.
**Figure S6:** Markers of main cell types in retinal organoids.
**Figure S7:** The expression of Müller cell‐specific genes in retinal organoids.
**Figure S8:** Expression of mitochondrial genes in photoreceptors.
**Figure S9:** The Maximal respiration and ATP production of MIO‐M1 cells transfected.
**Figure S10:** Impact of CLRN1 variants in MIO‐M1 cells on mitochondria, protein expression, and survival of 661W cells after 2‐day treatment.
**Figure S11:** Impact of CLRN1 variants in MIO‐M1 cells on mitochondrial function of 661W cells after 4‐day treatment.
**Table S1:** Antibodies used for immunofluorescent (IF) and Western blot (WB).

**Data S1:** Supporting Information.

## Data Availability

The data that support the findings of this study are available on request from the corresponding author. The data are not publicly available due to privacy or ethical restrictions.
