# Supplementary material for: CLRN1 Variants in Müller Cells Cause Mitochondrial Dysfunction in USH3A Retinal Organoids
Source: CNS Neurosci Ther. 2026 Aug 3;32(8):e71068. doi: 10.1002/cns.71068 (PMC13431285; doi:10.1002/cns.71068)
Supplement: Supplementary file 1 — Figure S1: Conservation analysis of the CLRN1 variants. Figure S2: mRNA levels of CLRN1 in muscles and HEK293 cells. Figure S3: Characterization of iPSCs for the patients. Figure S4: Clarin‐1 expression in fibroblasts and iPSC. Figure S5: The expression of Vimentin (VIM) in retinal organoids. Figure S6: Markers of main cell types in retinal organoids. Figure S7: The expression of Müller cell‐specific genes in retinal organoids. Figure S8: Expression of mitochondrial genes in photoreceptors. Figure S9: The Maximal respiration and ATP production of MIO‐M1 cells transfected. Figure S10: Impact of CLRN1 variants in MIO‐M1 cells on mitochondria, protein expression, and survival of 661W cells after 2‐day treatment. Figure S11: Impact of CLRN1 variants in MIO‐M1 cells on mitochondrial function of 661W cells after 4‐day treatment. Table S1: Antibodies used for immunofluorescent (IF) and Western blot (WB). [file CNS-32-e71068-s002.docx]

**CLRN1 Variants in Müller Cells Cause Mitochondrial Dysfunction in USH3A Retinal Organoids**

Rui Zhang^1,2^, Xinbo Ji^2^, Han Yu^1^, Jingwen Xu^2^, Yu Wang^3^, Ping Sun^3^, Yingxin Wang^2^, Yao Tang^2^, Zexin Zhan^2^, Yichang Jiao^2^, Didi Shan^2^, Pengfei Lin^2^, Dong-dong Wang^2^, Yuying Zhao^2^, Xianyang Liu^4^, Chuanzhu Yan^2, 5^, Jianqiao Li^1^, Mingfeng Li^6#^, Fuchen Liu^2#^, Shengping Hou^1,4#^

^1^Department of Ophthalmology, Qilu Hospital of Shandong University, Jinan, 250012, China;

^2^Department of Neurology, Research Institute of Neuromuscular and Neurodegenerative Diseases, Qilu Hospital of Shandong University, Shandong Key Laboratory of Mitochondrial Medicine and Rare Diseases, Jinan, 250012, China;

^3^Prenatal Diagnostic Center of Obstetrics and Department of Gynecology, Qilu Hospital of Shandong University, Jinan, 250012, China;

^4^Beijing Institute of Ophthalmology, Beijing Tongren Eye Center, Beijing Tongren Hospital, Capital Medical University, Beijing Ophthalmology & Visual Sciences Key Laboratory, Beijing, 100730, China;

^5^Qingdao Municipal Key Laboratory of Mitochondria Medicine Qingdao, 266035, China;

^6^Department of Pharmacology, Innovation center for Brain Medical Sciences, Tongji Medical College, Huazhong University of Science and Technology, The Key Laboratory for Drug Target Researches and Pharmacodynamic Evaluation of Hubei Province, Wuhan 430030, China

^#^ These authors contributed equally to this work.

Mingfeng Li, [mingfengli@hust.edu.cn](mailto:mingfengli@hust.edu.cn)

Fuchen Liu, fuchen.liu@email.sdu.edu.cn

Shengping Hou, sphou828@163.com

#
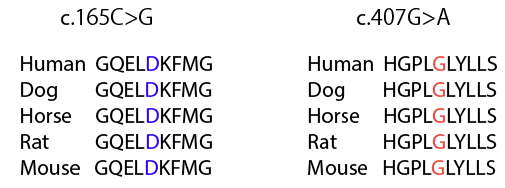


**Figure S1. Conservation analysis of the *CLRN1* variants.**

#
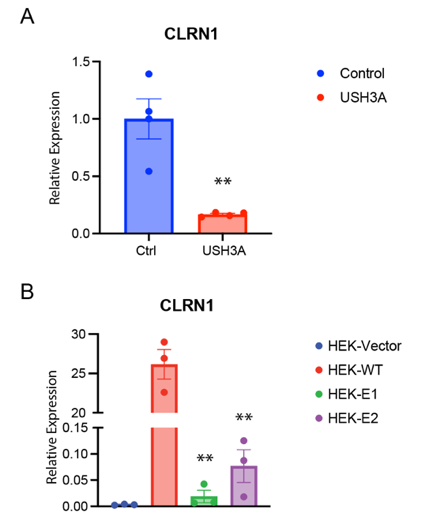


**Figure S2. mRNA levels of *CLRN1* in muscles and HEK293 cells**

A. mRNA levels of *CLRN1* in control and USH3A patient’s muscles. Statistical significance was evaluated by t-test. **P< 0.01. Data are shown as mean ± SEM.

B. mRNA levels of *CLRN1* in HEK cells transfected with vector, WT, E1 and E2. Statistical significance was evaluated by t-test. **P< 0.01. Data are shown as mean ± SEM.


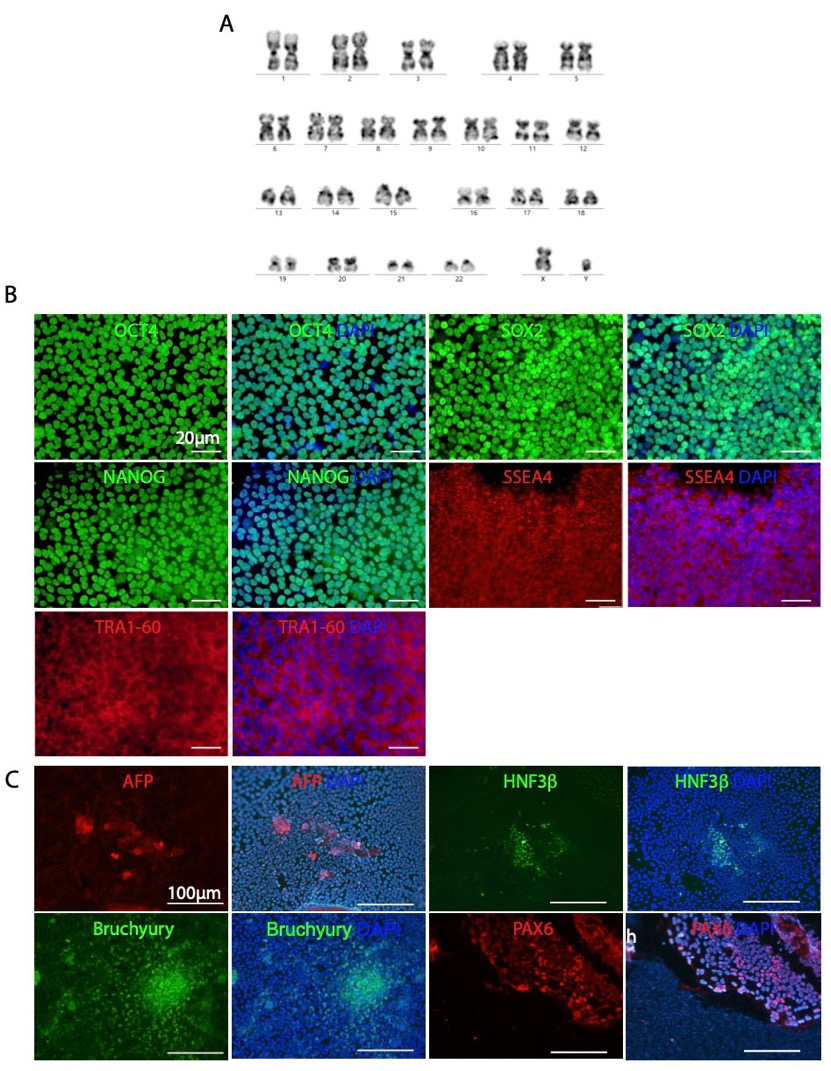


**Figure S3 Characterization of iPSCs for the patients**

A. Karyotype of the iPSCs.

B. Immunofluorescence of stem cell markers, OCT4, SOX2, NANOG, SSEA4 and TRA1-60 in iPSC. Scale bar: 20μm.

C. Immunofluorescence of markers of endoderm (AFP and HNF3β), mesoderm (Bruchyury) and ectoderm (PAX6) in embryoid bodies. Scale bar: 100μm.


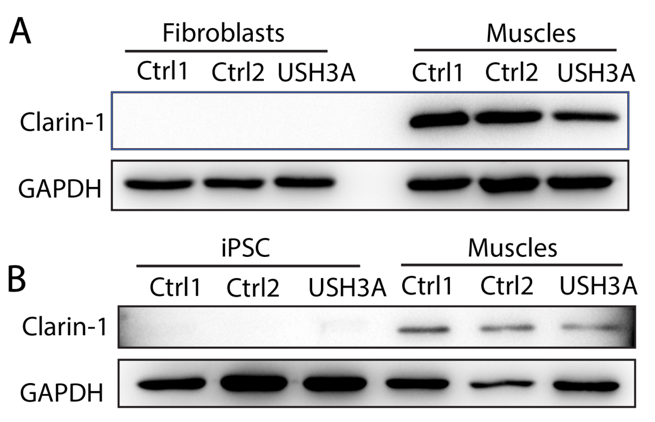


**Figure S4. Clarin-1 expression in fibroblasts and iPSC**

A. Clarin-1 expression in fibroblasts of control and the USH3A patient. Muscles were used as positive controls.

B. Clarin-1 expression in iPSCs of control and the USH3A patient. Muscles were used as positive controls.


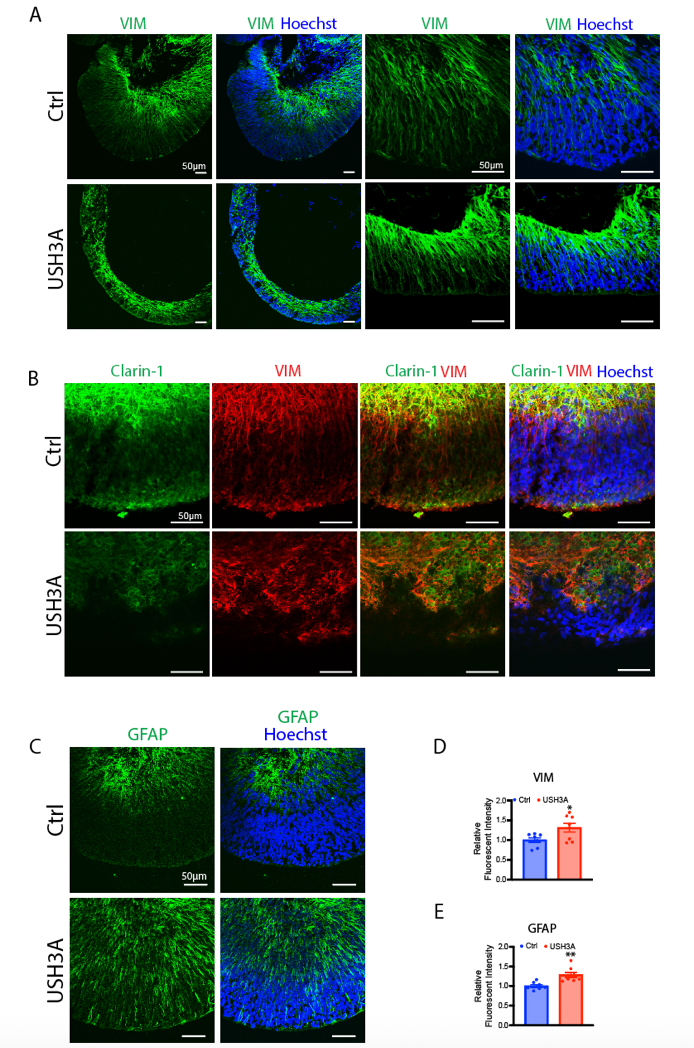


**Figure S5. The expression of Vimentin (VIM) in retinal organoids**.

A. Immunofluorescence of VIM in control and USH3A retinal organoids. Scale bar: 50μm.

B. Immunofluorescence of Clarin-1 and VIM in Ctrl and USH3A retinal organoids. Scale bar: 50μm.

C. Immunofluorescence of GFAP in Ctrl and USH3A retinal organoids. Scale bar: 50μm.

D-E. Quantification of the relative fluorescent intensity of VIM (D) and GFAP (E) in A and C. Numbers of samples showed as data points. Statistical significance was evaluated by t-test. **P< 0.01,*P< 0.05. Data are shown as mean ± SEM.


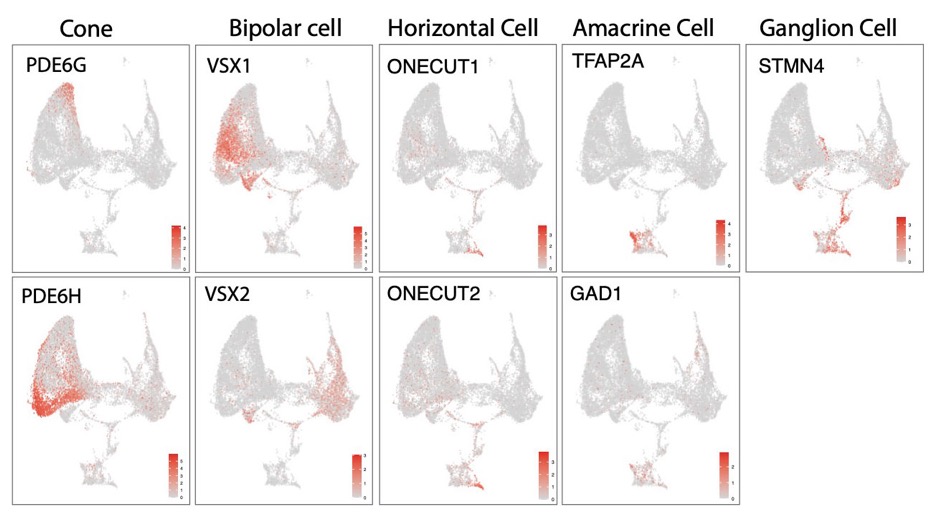


**Figure S6. Markers of main cell types in retinal organoids**

UMAP showing the expression of cone markers (PDE6G and PDE6H), bipolar cell markers (VSX1 and VSX2), horizontal cell markers (ONECUT1, ONECUT2), amacrine cell markers (TFAP2A and GAD1) and ganglion marker (STMN4) in the retinal organoids.


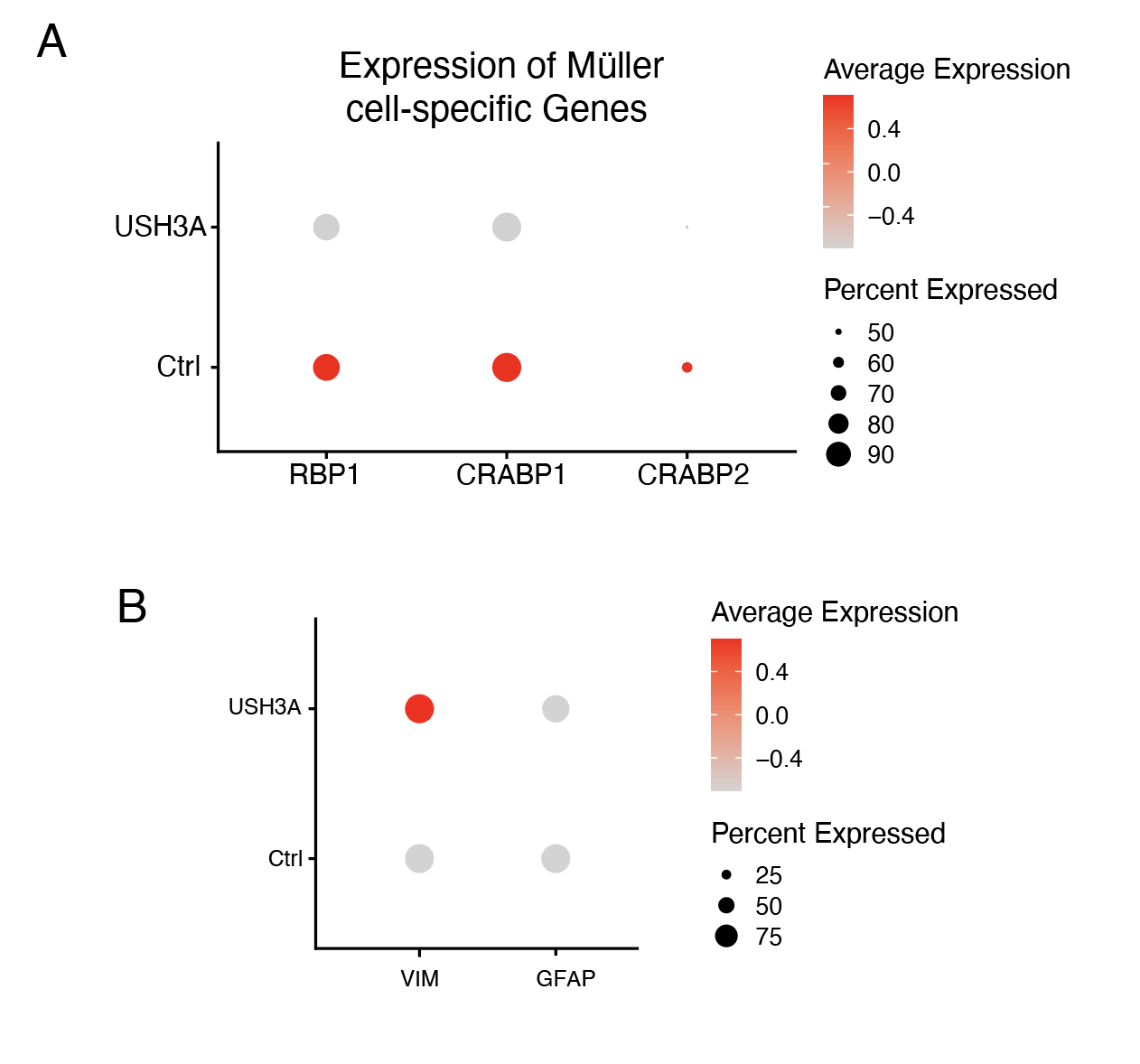


**Figure S7. The expression of Müller cell-specific genes in retinal organoids**

A. Bubble diagram of the expression of Müller cell-specific genes in Müller cells.

B. Bubble diagram of VIM and GFAP expression in Müller cells.


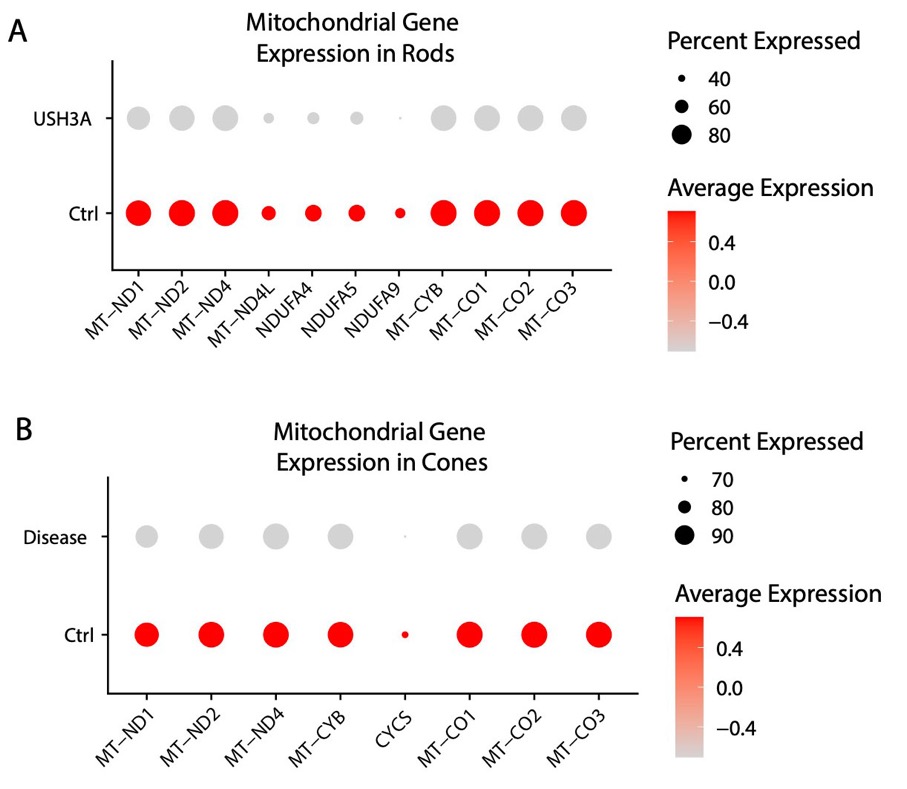


**Figure S8. Expression of mitochondrial genes in photoreceptors**

A. Bubble plot showing the expression of mitochondrial genes in control and the USH3A rods.

B. Bubble plot showing the expression of mitochondrial genes in control and the USH3A cones.


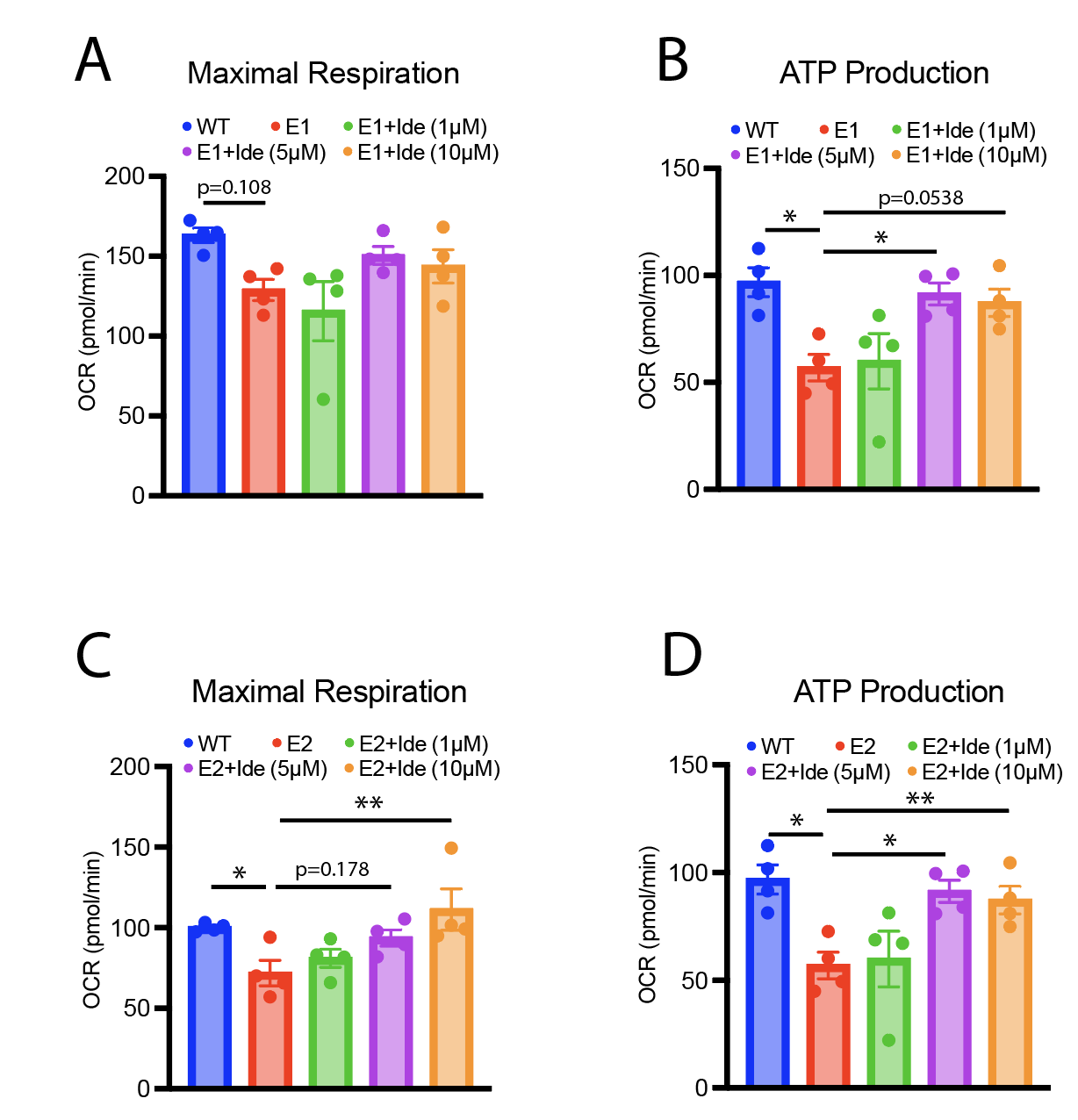


**Figure S9. The Maximal respiration and ATP production of MIO-M1 cells transfected with**

A-B. Maximal respiration and ATP production of MIO-M1 cells transfected with WT and E1, treated with 1, 5, 10μM Idebenone. Numbers of samples showed as data points. Statistical significance was evaluated by one-way ANOVA followed by Dunnett’s multiple comparisons test. *P< 0.05. Data are shown as mean ± SEM.

C-D. Maximal respiration and ATP production of MIO-M1 cells transfected with WT and E2, treated with 1, 5, 10μM Idebenone. Numbers of samples showed as data points. Statistical significance was evaluated by one-way ANOVA followed by Dunnett’s multiple comparisons test. **P< 0.01,*P< 0.05. Data are shown as mean ± SEM.


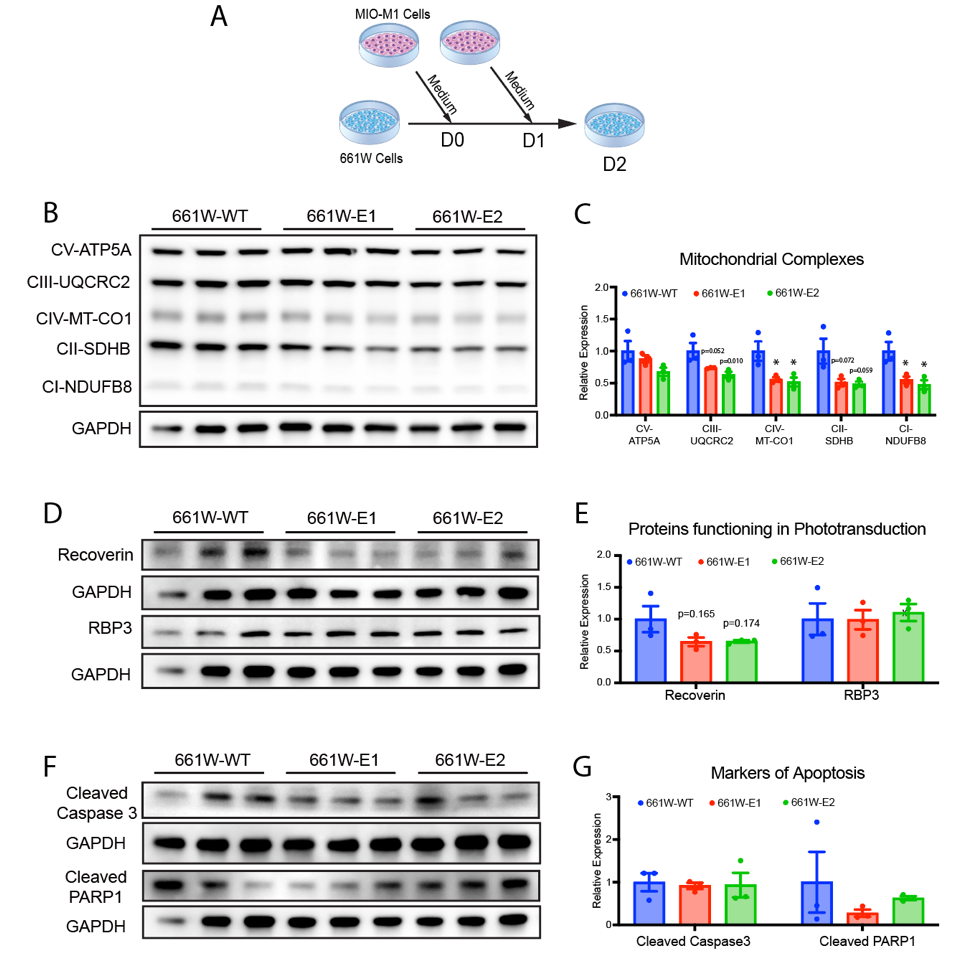


**Figure S10. Impact of *CLRN1* variants in MIO-M1 cells on mitochondria, protein expression, and survival of 661W cells after 2-day treatment.**

A. Schematic representation of the conditioned medium transfer experiment. MIO-M1 Müller cells were transfected with WT or *CLRN1* variants (E1, E2) for 48 hours, followed by medium collection. This medium was then applied to 661W photoreceptor-like cells, with daily medium refreshment for 2 days.

B, C. Western blot of mitochondrial complexes of 661W cells treated with medium of MIO-M1 cells transfected with WT, E1 and E2 for 2 days (B). CI, CII, CIII, CIV, CV: mitochondria complex I, II, III, IV, V. Quantification of mitochondrial complexes expression (C). Numbers of samples showed as date points. Statistical significance was evaluated by t-test. *P< 0.05. Data are shown as mean ± SEM.

D, E. Western blot analysis of photoreceptor markers, recoverin and RBP3, in 661W cells treated with medium from MIO-M1 cells after 2 days (D). Quantification of protein expression (E). Numbers of samples showed as date points. Statistical significance was evaluated by t-test. *P < 0.05. Data are shown as mean ± SEM.

F, G. Evaluation of apoptosis markers, cleaved caspase-3 and cleaved PARP-1, in 661W cells treated with medium from MIO-M1 cells containing WT, E1 or E2 for 2 days. (F). Quantification of protein expression (G). Numbers of samples showed as date points. Statistical significance was evaluated by t-test. *P< 0.05. Data are shown as mean ± SEM.


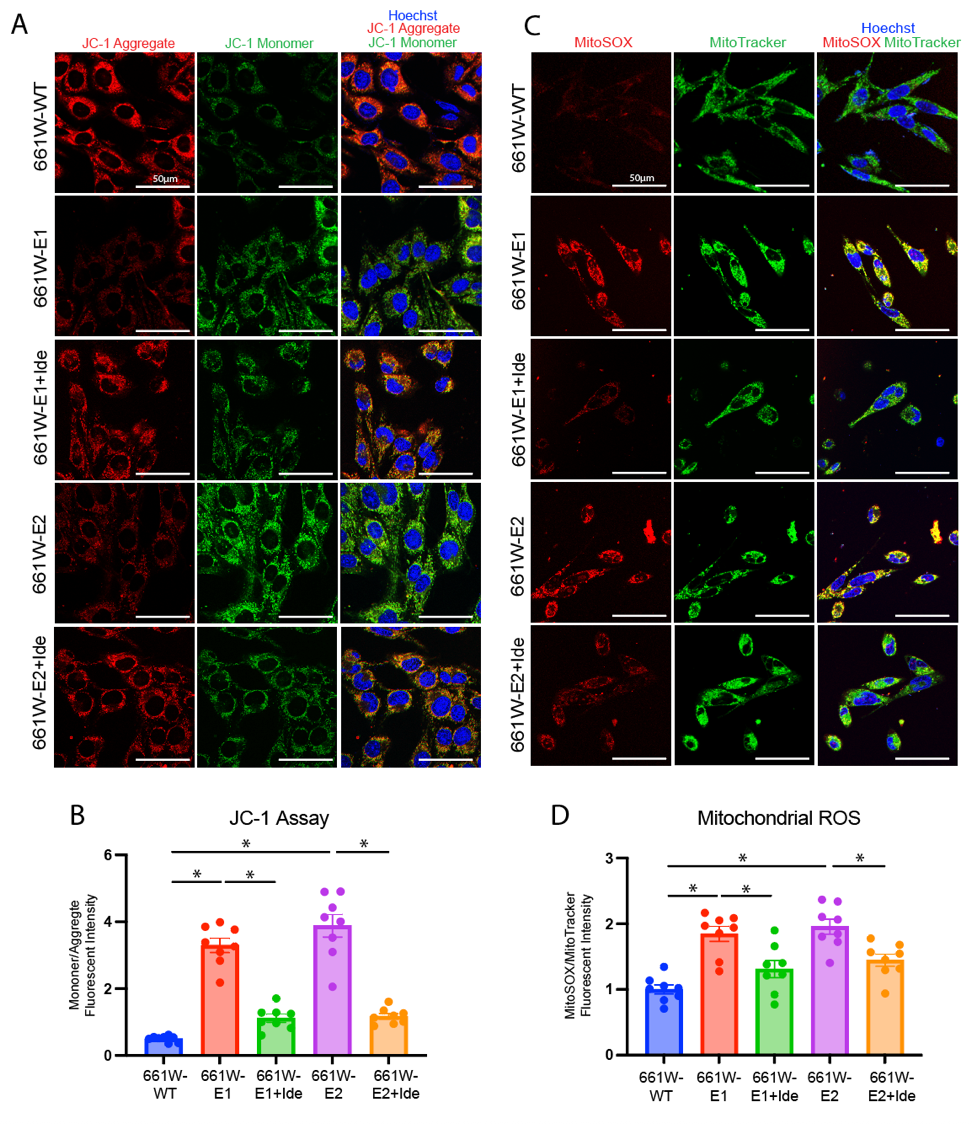


**Figure S11. Impact of *CLRN1* variants in MIO-M1 cells on mitochondrial function of 661W cells after 4-day treatment.**

A, B. JC-1 assay of 661W cells treated with medium of MIO-M1 cells transfected with WT, E1 and E2 for 2 days, treated with or without 5 μM idebenone (A). Scale bar: 50μm. Quantification of the fluorescence intensity ratio of JC-1 mononer to JC-1 aggregates (B). Statistical significance was evaluated by t-test. *P< 0.05. Data are shown as mean ± SEM.

C, D. MitoSOX and MitoTracker staining of 661W cells treated with medium of MIO-M1 cells transfected with WT, E1 and E2 for 2 days, treated with or without 5 μM idebenone (C). Scale bar: 50μm. Quantification of the fluorescence intensity ratio of MitoSOX stained area to MitoTracker stained area (D). Statistical significance was evaluated by t-test. *P< 0.05. Data are shown as mean ± SEM.

**Supplement Table 1 Antibodies used for immunofluorescent (IF) and Western blot (WB).**

| Target | Species | Manufacturer | Catalogue No. | Dilution (IF) | Dilution (WB) |
| --- | --- | --- | --- | --- | --- |
| AFP | Rabbit | ProteinTech | 14550–1-AP | 1:100 | - |
| Brachyury | Mouse | Santa Cruz | sc-374321 | 1:100 | - |
| Cleaved Caspase 3 | Rabbit | Cell Signaling | 9661 | 1:100 | 1:1000 |
| Cleaved PARP1 | Rabbit | Abclonal | A19612 | 1:100 | 1:2000 |
| CLRN1 | Rabbit | Invitrogen | PA5-70444 | - | 1:1000 |
| CLRN1 | Rabbit | ProteinTech | 26630-1-AP | 1:100 | - |
| CRABP1 | Rabbit | Abclonal | A5434 | 1:100 | - |
| CRABP2 | Rabbit | Abclonal | A6119 | 1:100 | - |
| CRALBP | Rabbit | Abcam | ab15051 | 1:100 | - |
| GFAP | Mous | ProteinTech | 60190-1-Ig | 1:100 | - |
| total OXPHOS antibody cocktail | Mouse | Abcam | ab110413 | - | 1:500 |
| Mt-Cyb | Rabbit | ProteinTech | 55090-1-AP | 1:100 | - |
| Mt-ND1 | Rabbit | Invitrogen | PA5-36493 | 1:100 | - |
| NANOG | Rabbit | ProteinTech | 14295–1-AP | 1:100 | - |
| OCT4 | Mouse | ProteinTech | 60242–1-Ig | 1:100 | - |
| PAX6 | Rabbit | ProteinTech | 12323–1-AP | 1:100 | - |
| PDE6G/H Antibody | Mouse | Santa Cruz | sc-166350 | 1:100 | - |
| RBP3 | Rabbit | Abclonal | A6403 | - | 1:2000 |
| Recoverin | Rabbit | Abclonal | A25214 | 1:100 | 1:5000 |
| Rhodopsin | Mouse | Abcam | ab5714 | 1:100 | - |
| SOX2 | Rabbit | ProteinTech | 11064–1-AP | 1:100 | - |
| SOX9 | Mouse | ProteinTech | 67439-1-Ig | 1:100 | - |
| SSEA4 | Mouse | Santa Cruz | sc-21704 | 1:100 | - |
| TRA-1–60 | Mouse | Santa Cruz | sc-21705 | 1:100 | - |
| TUBB3 | Mouse | ProteinTech | 66375–1-Ig | 1:100 | - |
| VIM | Rabbit | Cell Signaling | 5741 | 1:100 | - |
| VIM | Mouse | ProteinTech | 60330-1-Ig | 1:100 | - |
| VSX1 | Rabbit | ProteinTech | 23566-1-AP | 1:100 | - |
| Anti-mouse (AlexaFluor 488) | - | Invitrogen | A-21202 | 1:1000 | - |
| Anti-Mouse (AlexaFluor 594) | - | Invitrogen | A-21207 | 1:1000 | - |
| Anti-rabbit (AlexaFluor 488) | - | Invitrogen | A-21206 | 1:1000 | - |
| Anti-rabbit (AlexaFluor 594) | - | Invitrogen | A-21207 | 1:1000 | - |
