# Supplementary material for: CLRN1 Variants in Müller Cells Cause Mitochondrial Dysfunction in USH3A Retinal Organoids
Source: CNS Neurosci Ther. 2026 Aug 3;32(8):e71068. doi: 10.1002/cns.71068 (PMC13431285; doi:10.1002/cns.71068)

Fig 1N

CLRN1

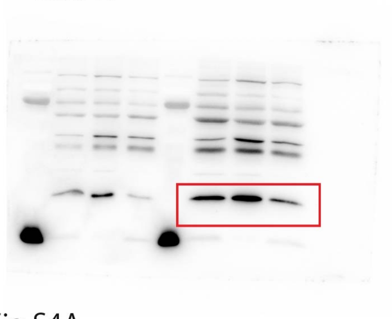

GAPDH

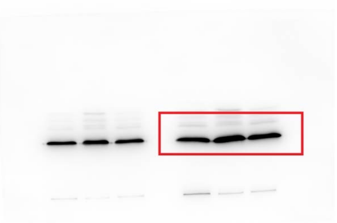

Fig S4A

CLRN1

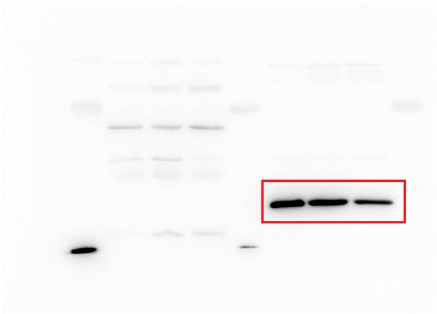

GAPDH

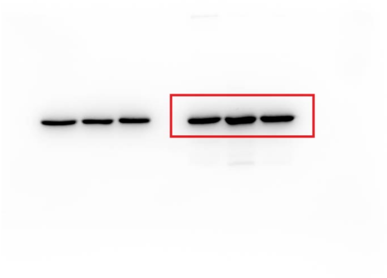

Fig S4B

CLRN1

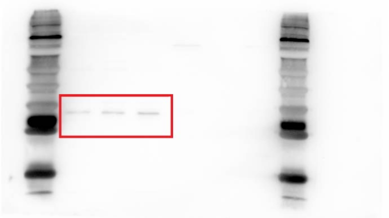

GAPDH

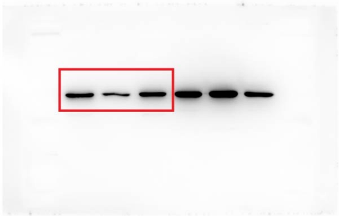

Fig 7
mt Complexes

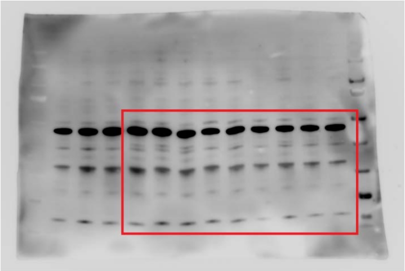

GAPDH

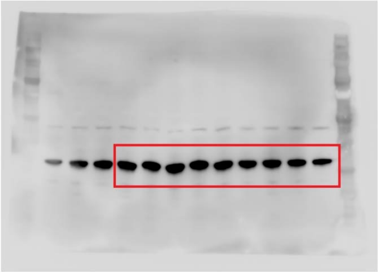

CLRN1

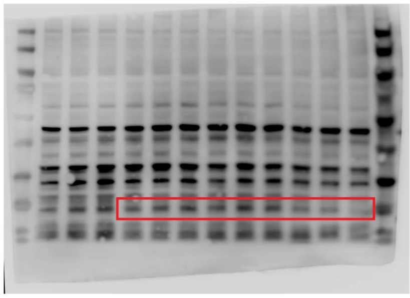

GAPDH

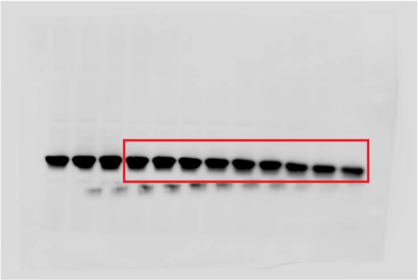

Fig 8 B OXPHOS

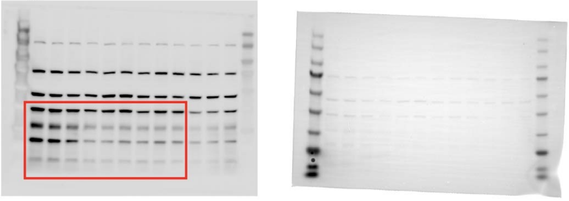

Fig 8 B GAPDH

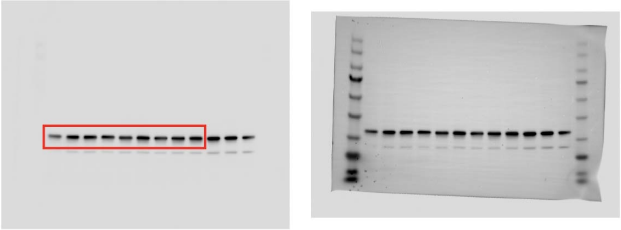

Fig 8 H RBP3

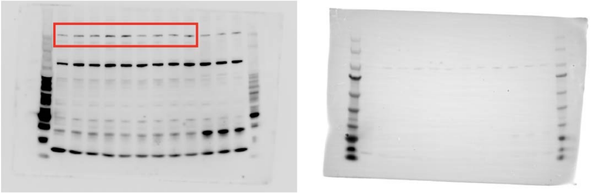

Fig 8 H GAPDH (RBP3)

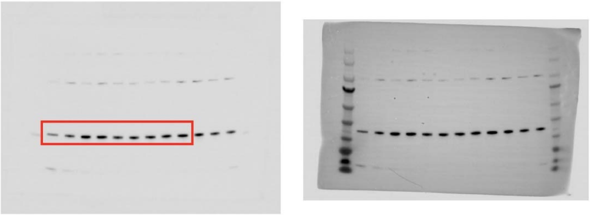

Fig 8 H Recoverin

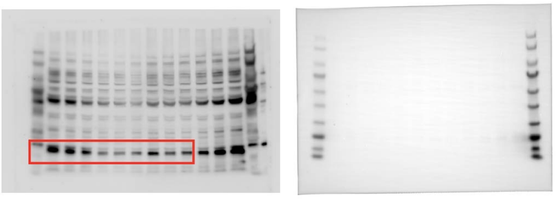

Fig 8 H GAPDH (Recoverin)

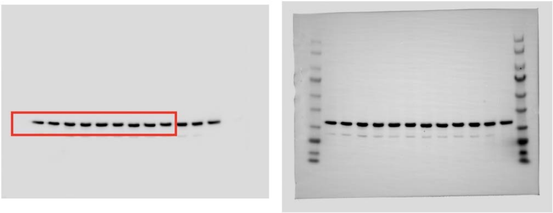

Fig 8 J Cleaved Caspase 3

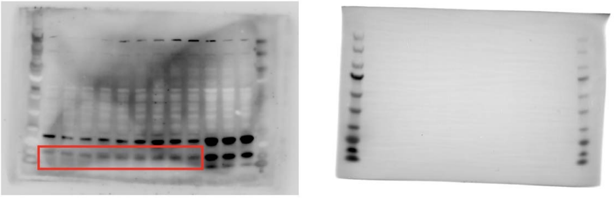

Fig 8 J Cleaved PARP1

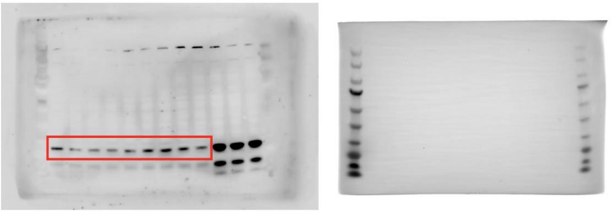

Fig 8 J GAPDH (Cleaved Caspase 3, Cleaved PARP1)

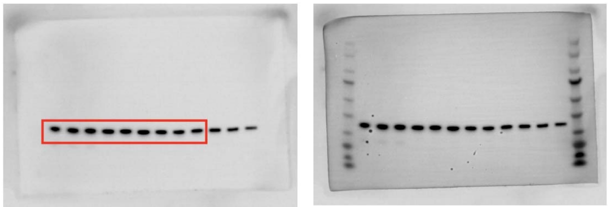

Fig S10 B OXPHOS

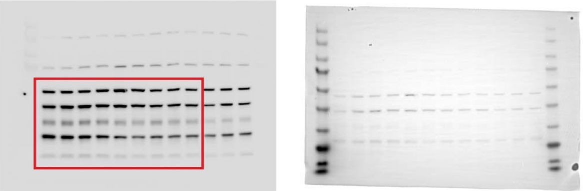

Fig S10 B GAPDH

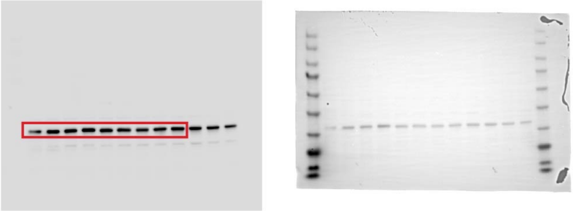

Fig S10 D RBP3-1-marker

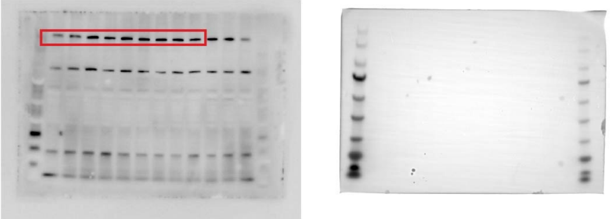

Fig S10 D GAPDH (RBP3)

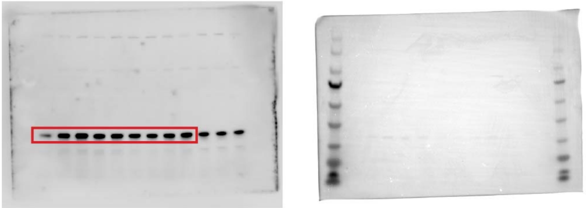

Fig S10 F Cleaved PARP1

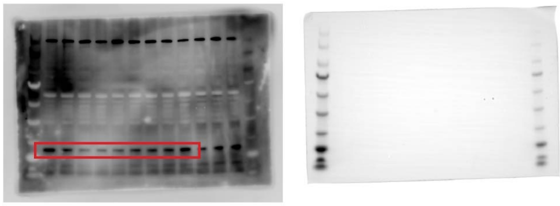

Fig S10 F GAPDH (Cleaved PARP1)

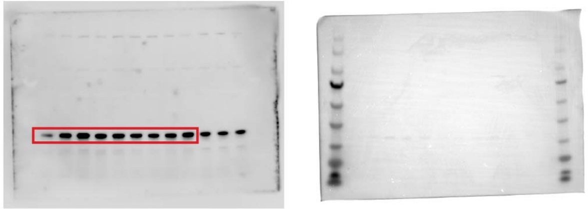

Fig S10 D Recoverin

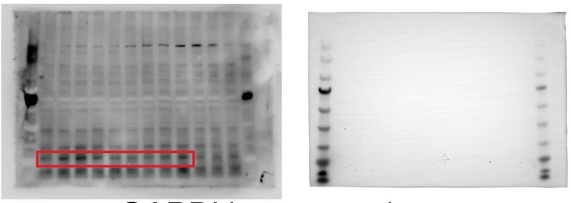

Fig S10 D GAPDH (Recoverin)

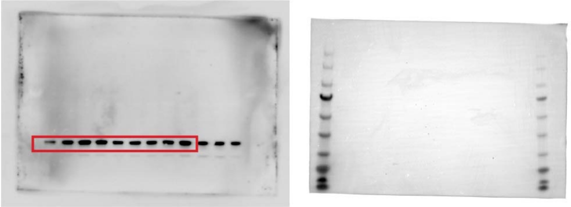

Fig S10 F Cleaved Caspase 3

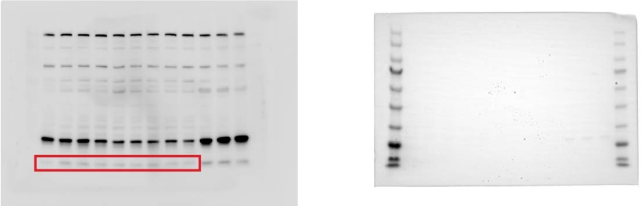

Fig S10 F GAPDH (Cleaved Caspase 3)

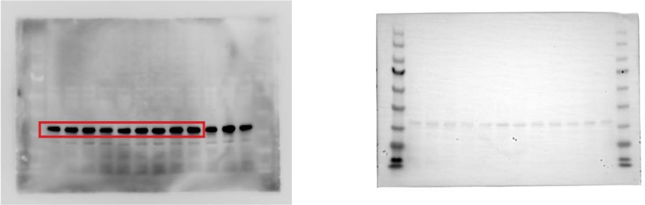

Supplement: Supplementary file 2 — Data S1: Supporting Information. [file CNS-32-e71068-s001.pdf]
